# Supplementary material for: Using phage display for rational engineering of a higher-affinity humanized 3’ phosphohistidine-specific antibody
Source: Commun Chem. 2025 Nov 27;8:381. doi: 10.1038/s42004-025-01768-9 (PMC12660843; doi:10.1038/s42004-025-01768-9)
Supplement: Supplementary file 1 — New supplementary information [file 42004_2025_1768_MOESM1_ESM.pdf]

Supplementary Figure legends

Fig. S1. Kabat numbering of rSC44 and hSC44 antibodies with associated protein expression and phage-display data. *(A-B)* IMGT nomenclature for *(A)* the V_L domain and *(B)* the V_H domain. *(C-D)* Kabat nomenclature for *(C)* the V_L domain and *(D)* the V_H domain. CDR positions and important framework positions necessary for humanization are colored as in Fig. 1. Positions in hSC44 that are the same as in rSC44 are left blank and differences are shown with the single-letter amino acid code. (E) Non-reducing SDS-PAGE of hSC44 and trastuzumab Fab and IgG protein. (F) Phage-ELISA was used to confirm hSC44 Fab (left) and scFv (right) displayed on phage as well as binding to 3-pTza. Phage were purified and concentrated to $1x10^{12}$ cfu/mL and serially diluted. FLAG indicates display levels of hSC44 Fab and scFv.

Fig. S2. Schematic of the workflow for generation and enrichment of pHis-containing peptides. Panel 1. Phosphoramidate Reaction**:** A degenerate histidine-containing peptide (Biotin-AGAGHAGAG) was reacted with potassium phosphoramidate. The reaction yielded products: non-phosphorylated, 1-pHis, 3-pHis and 1/3 di-pHis. Panel 2. Phosphohistidine Immunoprecipitation: The reaction was quenched and custom rSC1 and rSC44 antibody resins were used to preferentially enrich 1- pHis or 3-pHis containing peptide products, respectively. See Materials and Methods for a more detailed description of the protocol.

Fig. S3. Structure guided design of phage-displayed hSC44 libraries. The structure of rSC44 in complex with a 3-pTza peptide (PDB ID: 6X1V) was used to rationalize randomization strategies. Residue side chains are shown as sticks and colored as in Fig. 1. Residues that were proximal (within 10 Å) to the 3-pTza moiety were selected for randomization *(A-E)* Different residues in CDRs L1, L3, H1, H2, and H3 were randomized to maximize diversity and likelihood of identifying new combinations of specificity determining residues contacting 3-pHis. *(F)* Vernier zone and additional framework residues that were necessary for humanization of rSC44 were selected for randomization.

Fig. S4. Residues selected for randomization in hSC44 libraries. The amino acid sequences of rSC44 and hSC44 are shown using Kabat nomenclature. CDRs and Vernier Zone residues are highlighted and colored as in Fig. 1. Residues that were selected for randomization in each unique library are shown in bold underneath the sequence of hSC44. *(A)* Sequence of VL domain. *(B)* Sequence of VH domain.

Fig. S5. Characterization of hSC44 variants isolated from selections. *(A)* Variants of hSC44 were reformatted as IgG1 and expressed in Expi293 cells. The total protein yields were determined for each antibody with trastuzumab (black bar) and hSC44 (white bar) used as benchmarks. Variants with higher or lower yields than hSC44 are depicted with red or grey bars, respectively. *(B)* Non-reducing SDS-PAGE of purified hSC44 variant IgGs. Each variant IgG was loaded at 2.5 µg/well. *(C)* Lead hSC44 variants positively selected against 3-pTza or 3-pHis were assayed for binding to a 3-pHis peptide using ELISA. Data shown are an average of 4 replicates +/- SEM and displayed as fold change over hSC44 with hSC44 normalized to 1. *(D)* Lead hSC44 variants tested for binding to 3-pTza. 3-pHis, pTyr, pSer, and pSer peptides (biotin-Aminocaproic Acid-AGAGXAGAG; where "X" is 3-pTza, 3-pHis, pTyr, pSer or pThr) using ELISA. Each hSC44 variant IgG was used at 50 nM with varying concentrations of indicated peptides.

Fig. S6. BLI traces of hSC44 IgG variants assayed for binding to 3-pTza peptides. Biotinylated ACLYana-3-pTza was immobilized on the streptavidin biosensor tip and assayed for binding using varying concentrations of hSC44 Fab variants.

Fig. S7. BLI traces of hSC44 IgG variants assayed for binding to 3-pHis peptides. Biotinylated ACLYana-3-pHis (phosphorylated by phosphoramidate) was immobilized on the streptavidin biosensor tip and assayed for binding using varying concentrations of hSC44 Fab variants.

Fig. S8. BLI traces of hSC44 Fab variants assayed for binding to 3-pTza and 3-pHis peptides. Biotinylated ACLYana-3-pTza and ACLYana-3-pHis (phosphorylated by phosphoramidate) were immobilized on the streptavidin biosensor tip and assayed for binding using varying concentrations of hSC44 Fab variants. hSC44 Fab variants: ACLYana-3-pTza on left panel and hSC44 Fab variants: ACLYana-3-pHis on right panel.

Fig. S9. Specificity ELISA for hSC44 variants. hSC44 variant IgGs were tested for off-target binding against known antigenic molecules typically associated with poor antibody developability. See Materials and Methods for an in-depth description of the methodology.

Fig. S10. Effect of S1C substitutions on crystal packing. Replacement of the FG β-strand (198-204 Kabat numbering) of the light chain of the humanized antibody with residues from rabbit antibody (198-202 Kabat numbering) was used to enhance crystallization. The FG β-strand of the light chain formed hydrogen bond interactions with the constant domain of the heavy chain (206-210 Kabat numbering) from an adjacent protomer. These interactions propagated across protomers and mediated effective crystal packing.

Fig. S11. Effect of S1CE substitutions in the heavy chain elbow region on the elbow angle. Replacing SSASTKG with FNQIKG residues in the elbow region of the heavy chain shifted the elbow angles of Fabs to >160° (red) while Fabs without elbow mutations (green) stayed below this angle (Table S3). hSC44 Fabs are overlaid on their Cα carbons of the heavy chain variable region.

Fig. S12. Structural analysis of Vernier zone residue in hSC44. *(A)* Rabbit rSC44 (salmon red) and hSC44 (green) are overlaid on each other with a Ca RMSD of 0.75 $Å^2$. *(B)* Vernier residue $R46^{L}$ interacts with $N99^{H}$ and $P100^{H}$ residues on CDR-H3 loop which in turn interacts with phosphoryl group of 3-pTza. Note that Vernier zone residues are shown in green text. *(C)* $F34^{H}$ is oriented towards the core of the antibody and forms hydrophobic interactions with residues from several framework regions of the heavy chain and $K94^{H}$ from CDR-H3 that interacts with phosphoryl group. *(D)* Two patches of substitutions in the heavy chain framework region enhanced the structure and function of the hSC44 Fab. $H47^{H}$ and $Y50^{H}$ at the interface of heavy and light chains interact with $Y96^{L}$, which is essential for phosphoryl recognition. The ^{61}SWAK^{64} further stabilized the ^{47}HIGY^{50} region in CDR-H2 and the heavy framework region 3.

Fig. S13. Structural overlay of hSC44 variants. Fabs are overlaid based on the Cα carbons of their heavy chain variable region. Superposed hSC44 (green), hSC44.20 (orange) and hSC44.20.N32F^{L} (blue) have Ca RMSDs less than <1 Å in complex with *(A)* 3-pTza (magenta) or *(B)* 3-pHis (red) peptides.

Fig. S14. **Contact analysis between WT and N32F hSC44.20 and 3-pHis**. ***(A)*** The time-dependent variation in the total number of contacts for 3-pHis with (Orange) WT and (Blue) mutant (N32F) hSC44.20 in their respective environments. The black dashed line represents the average number of contacts between 3-pHis and the Fab region of the antibody over time. ***(B)*** Analysis of the types of contacts and their interaction fractions for (left) hSC44.20 with 3-pHis and (right) hSC44.20.N32F^{L} with 3-pHis. The interactions presented in panel B are those that are found >50% of the time during the 20 ns simulations. The π-π interaction involving the mutation (N32F^{L}) with 3-pHis are outlined with a black dotted line in **Figure 1B** (bottom). This stable interaction is present ~75% of the time during the 20 ns simulation, but it is not found in the structure of WT hSC44.20 with 3-pHis (left). In the Y-axis, "H" refers to the heavy-chain and "L" refers to the light-chain. Superscripts "A" and "B" refer to different chains in the protein structure.

Fig. S15. *(A)* Effect of N32F mutation on 3-pHis. *(B)* Electrostatic potential of 3-pHis and Phe32 showing the T-shaped π-π interactions. The color bar shows the change in charges across electron density. The black dashed line shows the T-shaped π-π interactions in each plot.

Fig. S16. Thermodynamic cycles employed for MD-based FEP simulations were utilized to compute the changes in binding free energy resulting from residue mutations in the antibody binding process. We examined the changes in the binding free energy caused by the N32F mutation in the binding of 3-pHis by using FEP (free energy perturbation) to calculate the relative binding free energy $\Delta\Delta G^{WT\rightarrow M}_{bind}$ for 3-pHis. In the thermodynamic cycle, the relative binding free energy $\Delta\Delta G^{WT\rightarrow M}_{bind}$ can be obtained by the two vertical legs, instead of simulating the physical binding process.

Fig. S17. Sequence specificity assays of rSC44-8 and hSC44 variants. Thermal shift assay of rSC44-8 (A), hSC44.20 (B) and hSC44.20.N32F^{L} (C) with 3-pTza peptides. Biolayer interferometry assay of rSC44-8 (D), hSC44.20 (E) and hSC44.20.N32F^{L} (F) variants with pHis peptides. Biotinylated peptides from pHis substrate proteins were phosphorylated by phosphoramidate and immobilized on the streptavidin biosensor tip.

Fig. S18. Differences in CDR loop orientations of hSC44 variants complexed with 3-pTza and 3-pHis peptides. Fabs are overlaid on the basis of the Cα carbons of their heavy chain variable regions. The 3-pTza (magenta) and 3-pHis (red) peptides bound to *(A)* hSC44 CDRs (green), *(B)* hSC44.20 CDRs (orange), and *(C)* hSC44.20.N32F^{L} CDRs (blue).

Fig. S19. hSC44.20.N32F^{L} in complex with 3-pTza and 3-pHis peptides. *(A-B)* Omit maps (F_O-F_C map) of *(A)* hSC44.20.N32F^{L} Fab (blue) in complex with 3-pTza peptide (magenta) and *(B)* hSC44.20.$N32^{L}$ (blue)

in complex with 3-pHis peptide (red). The bound peptide is contoured at 1.0 sigma and the CDR loops are represented as cartoon. The sequence of the peptide used for crystallization is shown below and the residues in red font have interpretable electron density. *(C-D)* Residues from CDRs H1, H2, H3, L1 and L3 of hSC44.20.N32F^L are making direct and water-mediated hydrogen bond interactions with the phosphoryl group of the 3-pTza peptide *(C)* or the 3-pHis peptide *(D)*. A coordinated water molecule (grey sphere) is conserved in both complexes. Residues depicted in red font are substitutions that are different from hSC44. *(E-F)* hSC44.20.N32F^L interactions with the peptide backbone of either *(E)* 3-pTza peptide or *(F)* 3-pHis peptide.

Fig. S20. Differences in triazolyl and imidazole ring interactions in hSC44.20.N32F^L. *(A)* Structures of 3-pTza and 3-pHis molecules. The nitrogen atoms that undergo phosphorylation in 3-pHis and the corresponding atoms in the triazolyl ring are marked in red. *(B)* hSC44.20.N32F^L makes direct and water-mediated (grey sphere) interactions with nitrogen 2 and nitrogen 3 atoms of the triazolyl moiety of the peptide through residues in CDRs H1, H2, H3, L1, and L3. *(C)* hSC44.20.N32F^L makes direct and water-mediated interactions with the imidazole moiety of 3-pHis through residues in CDRs H1, H2, L1, and L3. Interactions from CDR-H3 are absent due to the presence of a carbon atom in the imidazole ring position corresponding to position of nitrogen 3 in the triazolyl ring.

Fig. S21. Time-dependent variation in the total number of contacts for 3-pHis (Orange) and 3-pTza (Green) with their surroundings (criteria detailed in the computational methods section). The black dashed line represents the average number of contacts over time.

Fig. S22. QM/MM analysis of 3-pHis and 3-pTza. (A) Key atomic level structural differences near PO^{3-} between (left) 3-pHis (Backbone carbon atoms indicated with green) and (right) 3-pTza (Backbone carbon atoms indicated with white) antigens. (B) Electrostatic potential surface of (left) 3-pHis and (right) 3-pTza, showing Mulliken charges [18] on the O atom of PO_3^- from QM analysis (charges are in atomic units (au); the color bar indicates charge variations across the electron density. (C) Highest occupied molecular orbital (HOMO) and lowest unoccupied molecular orbital (LUMO) (iso-surface value 0.05 au) and corresponding energies of (left) 3-pHis and (right) 3-pTza from natural bond orbital (NBO) population analysis using QM; a lower HOMO-LUMO gap indicates that 3-pTza is more chemically active towards binding than 3-pHis. (D) Electrostatic potential surface of (left) 3-pHis and (right) 3-pTza with their surrounding environment from QM/MM analysis; the color bar shows charge variations across the electron density. The antigen with N-terminal histidine phosphorylation (N-PO_3^-, 3-pHis) and C-terminal phosphohistidine analog (C-PO_3^-, 3-pTza), facilitating binding to the Fab region, is shown with black dotted circles throughout the figure.

Fig. S23. Unliganded structures of hSC44 variants. *(A)* Differences in CDR loop orientation across unliganded structures of hSC44 (green), hSC44.20 (orange) and hSC44.20.N32F^L (blue) Fabs. *(B)* Comparison of CDR loop orientation in hSC44.20.N32F^L (blue) in complex with 3-pTza peptide (magenta) or 3-pHis peptide (red) or in unliganded state. *(C-F)* Hydrogen bond interactions of *(C)* sulfate ion, *(D)* phosphate ion, *(E)* HEPES, and *(F)* citrate ion bound to hSC44.20.N32F^L, hSC44.20, hSC44.20.N32F^L, or hSC44 Fabs, respectively.

Fig. S24. Structure of unliganded hSC44.20 Fab. *(A)* hSC44.20 Fab (orange) makes crystal contacts with an adjacent protomer (gray). The CDR-L2 loop and the epitope-binding region are marked with red and grey circles respectively. *(B)* The CDRs of unliganded hSC44.20 Fab bound the sidechain of residue E1^H of an adjacent protomer.

Fig. S1.

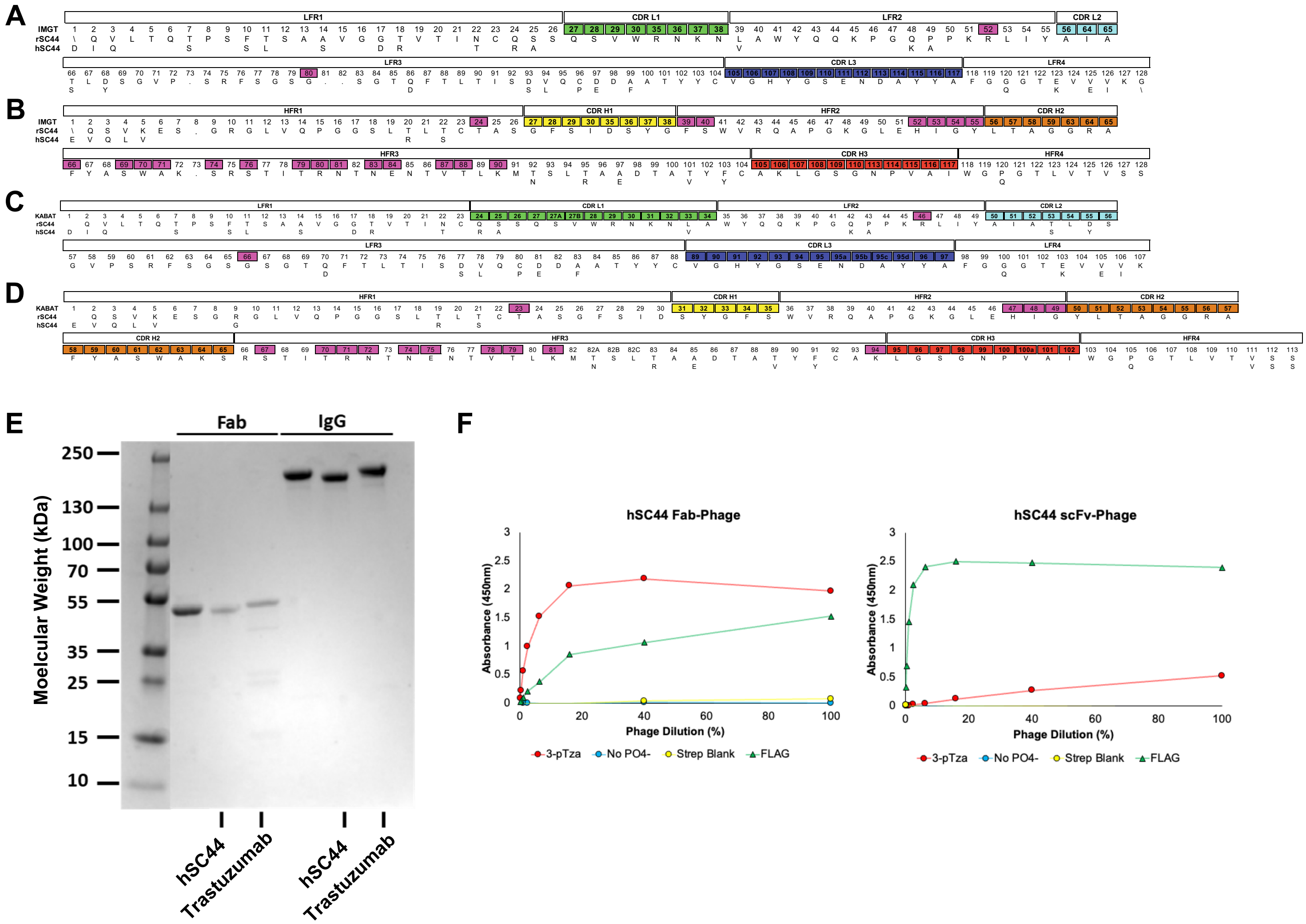
A
B
C
D
IMGT
rSC44
hSC44
KABAT
LFR1
CDR L1
LFR2
CDR L2
LFR3
CDR L3
LFR4
HFR1
CDR H1
HFR2
CDR H2
HFR3
CDR H3
HFR4
E
Fab
IgG
Moelcular Weight (kDa)
250
130
100
70
55
35
25
15
10
hSC44
Trastuzumab
hSC44
Trastuzumab
F
hSC44 Fab-Phage
hSC44 scFv-Phage
Absorbance (450nm)
Phage Dilution (%)
3-pTza
No PO4-
Strep Blank
FLAG

Fig. S2.

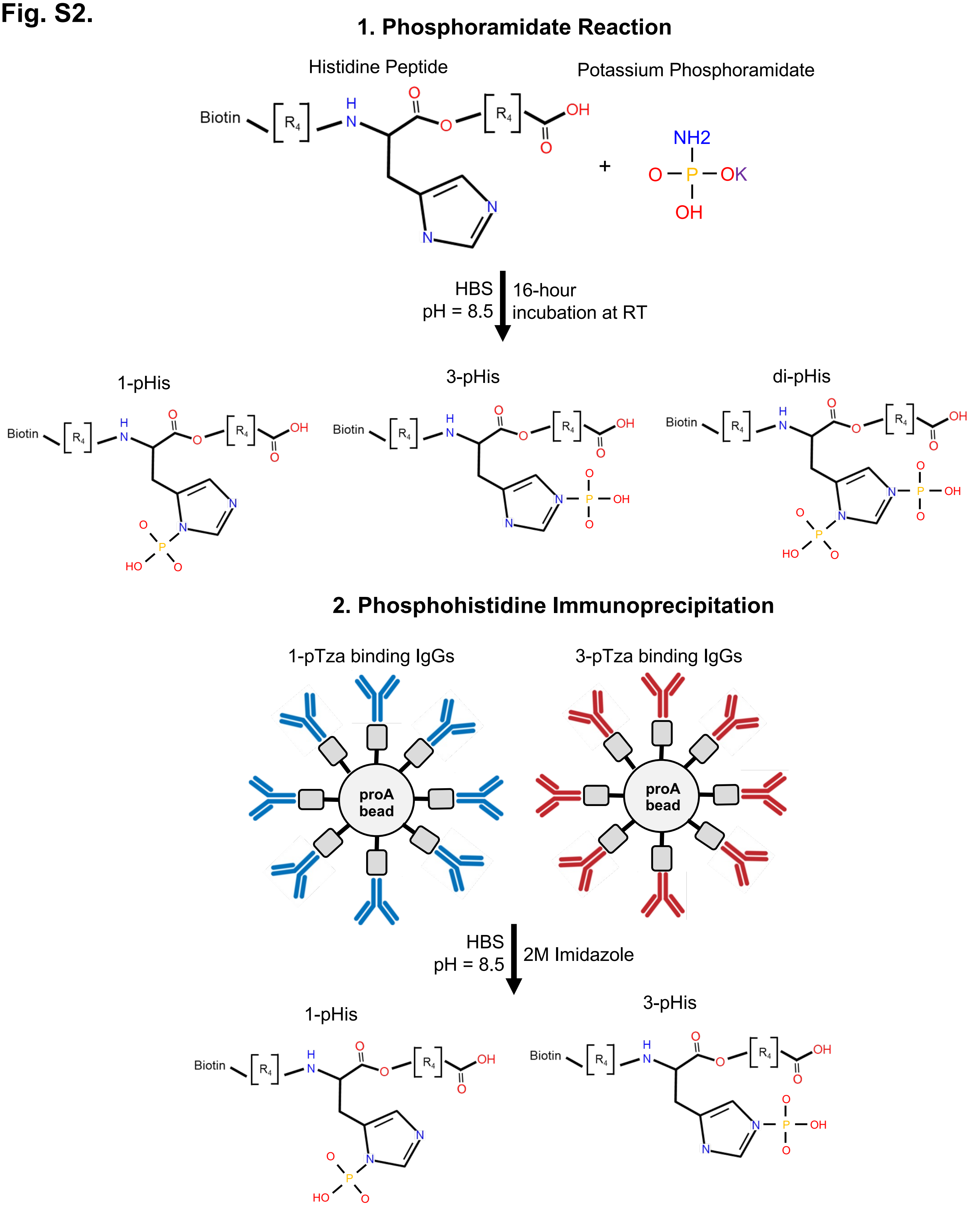
1. Phosphoramidate Reaction
Histidine Peptide
Potassium Phosphoramidate
Biotin
R4
NH2
OK
OH
HBS
pH = 8.5
16-hour
incubation at RT
1-pHis
3-pHis
di-pHis
2. Phosphohistidine Immunoprecipitation
1-pTza binding IgGs
3-pTza binding IgGs
proA
bead
HBS
pH = 8.5
2M Imidazole
1-pHis
3-pHis

Fig. S3.

A

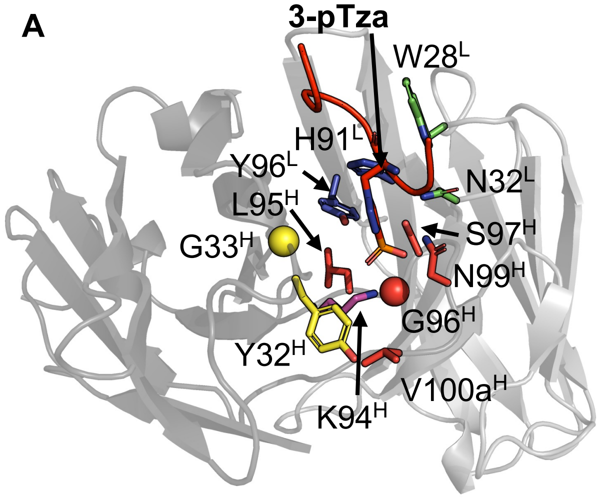

Library 1 Size: **2x10^{9}**

B

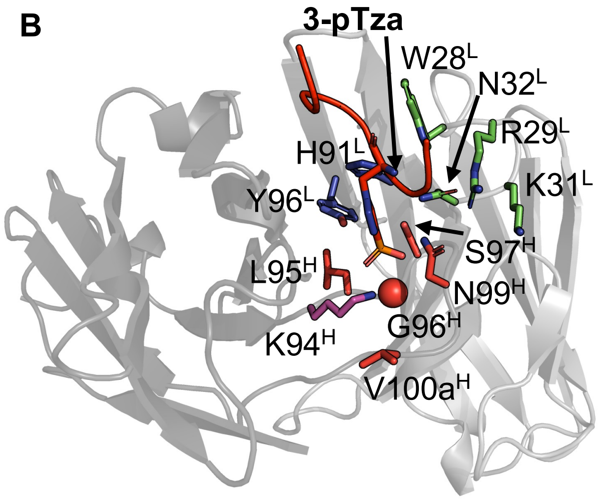

Library 2 Size: **2x10^{9}**

C

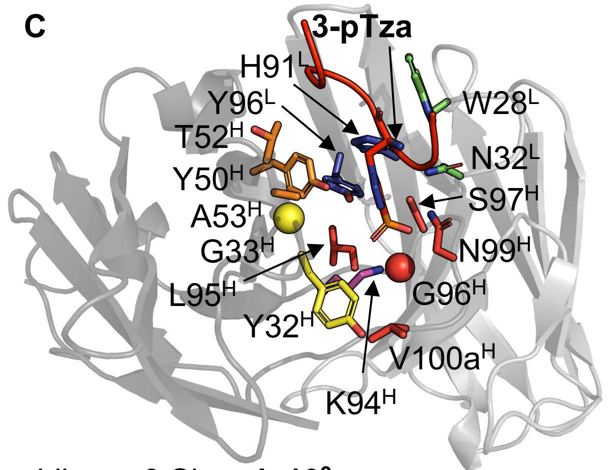

Library 3 Size: **4x10^{9}**

D

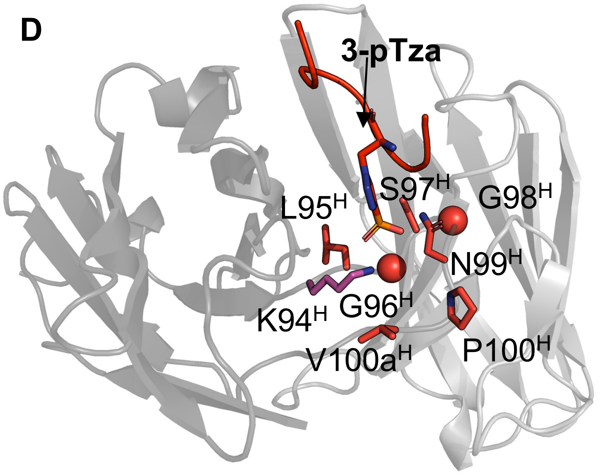

Library 4 Size: **2x10^{10}**

E

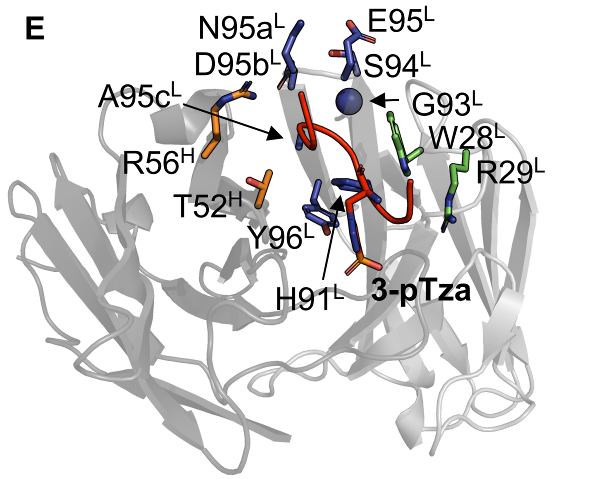

Library 5 Size: **2x10^{9}**

F

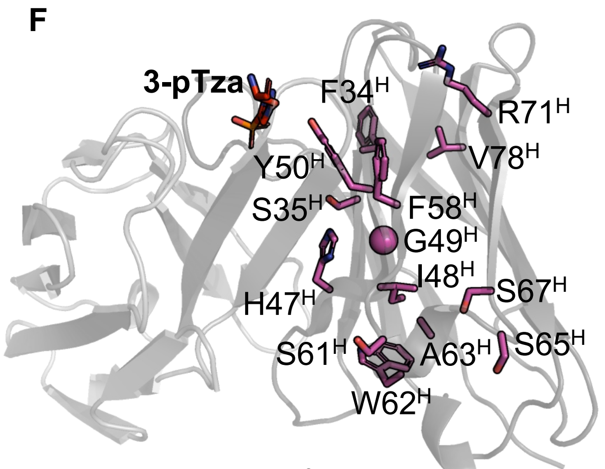

Library 6 Size: **3x10^{9}**

Fig. S4.

A

LFR1

		1	2	3	4	5	6	7	8	9	10	11	12	13	14	15	16	17	18	19	20	21	22	23
	KABAT	1	2	3	4	5	6	7	8	9	10	11	12	13	14	15	16	17	18	19	20	21	22	23
Scaffold	rSC44		Q	V	L	T	Q	T	P	S	F	T	S	A	A	V	G	G	T	V	T	I	N	C
Scaffold	hSC44	D	I	Q				S			S	L			S			D	R				T	
Library	hSC44 1																							
Library	hSC44 2																							
Library	hSC44 3																							
Library	hSC44 4																							
Library	hSC44 5																							
Library	hSC44 6																							

CDR L1

	KABAT	24	25	26	27	27A	27B	28	29	30	31	32	33	34
Scaffold	rSC44	Q	S	S	Q	S	V	W	R	N	K	N	L	A
Scaffold	hSC44	R	A										V	
Library	hSC44 1							**W**				**N**		
Library	hSC44 2							**W**	**R**		**K**	**N**		
Library	hSC44 3							**W**				**N**		
Library	hSC44 4													
Library	hSC44 5							**W**	**R**					
Library	hSC44 6													

LFR2

	KABAT	35	36	37	38	39	40	41	42	43	44	45	46	47	48	49
Scaffold	rSC44	W	Y	Q	Q	K	P	G	Q	P	P	K	R	L	I	Y
Scaffold	hSC44								K	A						
Library	hSC44 1–6															

CDR L2

	KABAT	50	51	52	53	54	55	56
Scaffold	rSC44	A	I	A	T	L	D	S
Scaffold	hSC44				S		Y	
Library	hSC44 1–6							

LFR3

	KABAT	57	58	59	60	61	62	63	64	65	66	67	68	69	70	71	72	73	74	75	76	77	78	79	80	81	82	83	84	85	86	87	88
Scaffold	rSC44	G	V	P	S	R	F	S	G	S	G	S	G	T	Q	F	T	L	T	I	S	D	V	Q	C	D	D	A	A	T	Y	Y	C
Scaffold	hSC44														D							S	L		P	E		F					
Library	hSC44 1–6																																

CDR L3

	KABAT	89	90	91	92	93	94	95	95a	95b	95c	95d	96	97
Scaffold	rSC44	V	G	H	Y	G	S	E	N	D	A	Y	Y	A
Scaffold	hSC44													
Library	hSC44 1			**H**									**Y**	
Library	hSC44 2			**H**									**Y**	
Library	hSC44 3			**H**									**Y**	
Library	hSC44 4													
Library	hSC44 5			**H**		**G**	**S**	**E**	**N**	**D**	**A**		**Y**	
Library	hSC44 6													

LFR4

	KABAT	98	99	100	101	102	103	104	105	106	107
Scaffold	rSC44	F	G	G	G	T	E	V	V	V	K
Scaffold	hSC44			Q			K		E	I	
Library	hSC44 1–6										

B

HFR1

	KABAT	1	2	3	4	5	6	7	8	9	10	11	12	13	14	15	16	17	18	19	20	21	22	23	24	25	26	27	28	29	30
Scaffold	rSC44		Q	S	V	K	E	S	G	R	G	L	V	Q	P	G	G	S	L	T	L	T	C	T	A	S	G	F	S	I	D
Scaffold	hSC44	E	V	Q	L	V				G										R		S									
Library	hSC44 1–6																														

CDR H1

	KABAT	31	32	33	34	35
Scaffold	rSC44	S	Y	G	F	S
Scaffold	hSC44					
Library	hSC44 1					
Library	hSC44 2		**Y**	**G**		
Library	hSC44 3					
Library	hSC44 4		**Y**	**G**		
Library	hSC44 5					
Library	hSC44 6				**F**	**S**

HFR2

	KABAT	36	37	38	39	40	41	42	43	44	45	46	47	48	49
Scaffold	rSC44	W	V	R	Q	A	P	G	K	G	L	E	H	I	G
Scaffold	hSC44														
Library	hSC44 1–5														
Library	hSC44 6												**H**	**I**	**G**

CDR H2

	KABAT	50	51	52	53	54	55	56	57	58	59	60	61	62	63	64	65
Scaffold	rSC44	Y	L	T	A	G	G	R	A	F	Y	A	S	W	A	K	S
Scaffold	hSC44																
Library	hSC44 1																
Library	hSC44 2																
Library	hSC44 3			**T**	**A**												
Library	hSC44 4																
Library	hSC44 5			**T**				**R**									
Library	hSC44 6	**Y**								**F**			**S**	**W**	**A**		**S**

HFR3

	KABAT	66	67	68	69	70	71	72	73	74	75	76	77	78	79	80	81	82	82A	82B	82C	83	84	85	86	87	88	89	90	91	92	93	94
Scaffold	rSC44	R	S	T	I	T	R	N	T	N	E	N	T	V	T	L	K	M	T	S	L	T	A	A	D	T	A	T	Y	F	C	A	K
Scaffold	hSC44																		N			R		E				V		Y			
Library	hSC44 1																																**K**
Library	hSC44 2																																**K**
Library	hSC44 3																																**K**
Library	hSC44 4																																**K**
Library	hSC44 5																																
Library	hSC44 6		**S**				**R**							**V**																			

CDR H3

	KABAT	95	96	97	98	99	100	100a	101	102
Scaffold	rSC44	L	G	S	G	N	P	V	A	I
Scaffold	hSC44									
Library	hSC44 1	**L**	**G**	**S**		**N**		**V**		
Library	hSC44 2	**L**	**G**	**S**		**N**		**V**		
Library	hSC44 3	**L**	**G**	**S**		**N**		**V**		
Library	hSC44 4	**L**	**G**	**S**	**G**	**N**	**P**	**V**		
Library	hSC44 5									
Library	hSC44 6									

HFR4

	KABAT	103	104	105	106	107	108	109	110	111	112	113
Scaffold	rSC44	W	G	P	G	T	L	V	T	V	S	S
Scaffold	hSC44			Q						V	S	S
Library	hSC44 1–6											

Fig. S5.

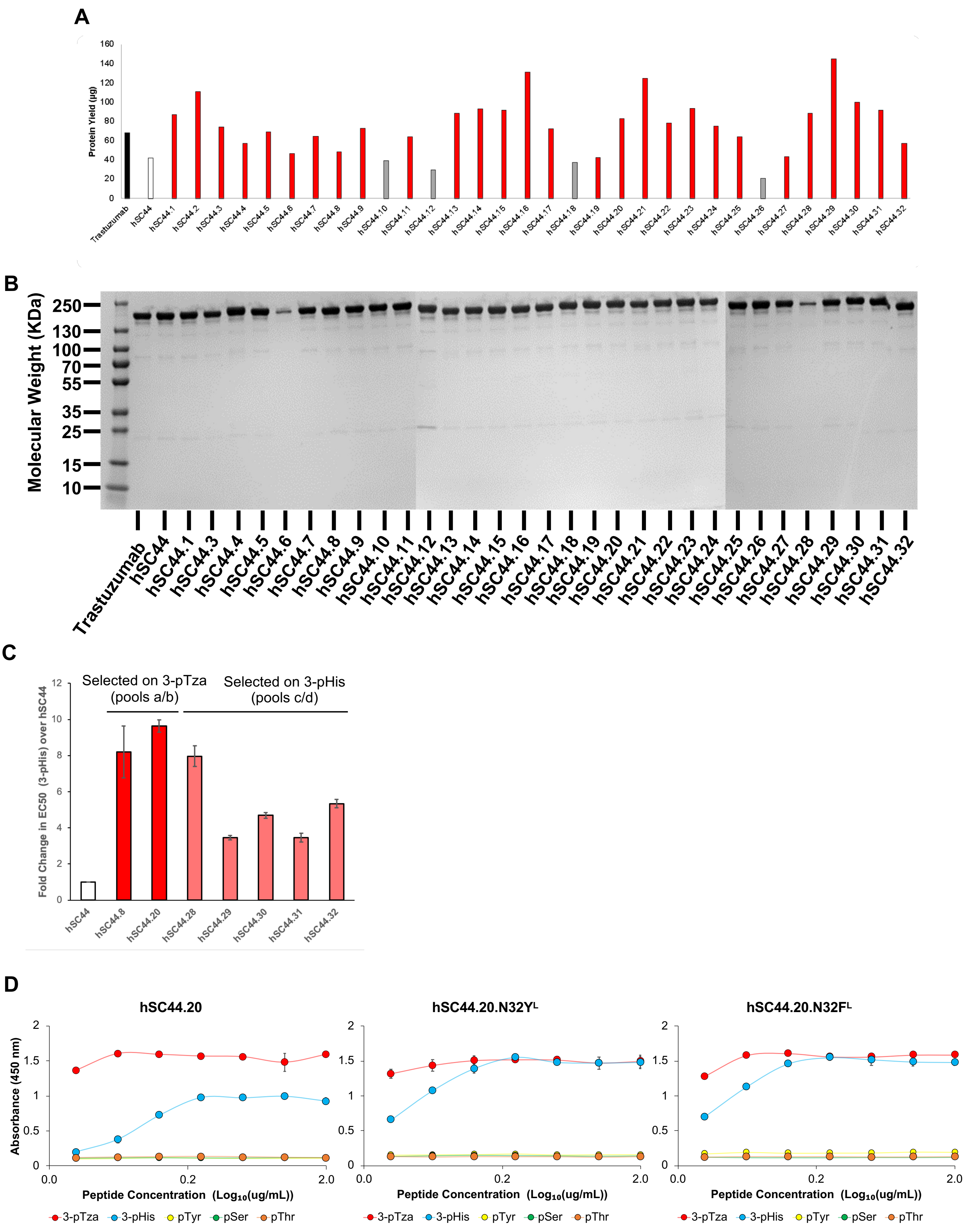
A
Protein Yield (µg)
Trastuzumab
hSC44
B
Molecular Weight (KDa)
250
130
100
70
55
35
25
15
10
C
Selected on 3-pTza (pools a/b)
Selected on 3-pHis (pools c/d)
Fold Change in EC50 (3-pHis) over hSC44
hSC44
hSC44.8
hSC44.20
hSC44.28
hSC44.29
hSC44.30
hSC44.31
hSC44.32
D
hSC44.20
hSC44.20.N32Y^L
hSC44.20.N32F^L
Absorbance (450 nm)
Peptide Concentration (Log10(ug/mL))
3-pTza
3-pHis
pTyr
pSer
pThr

Fig. S6.

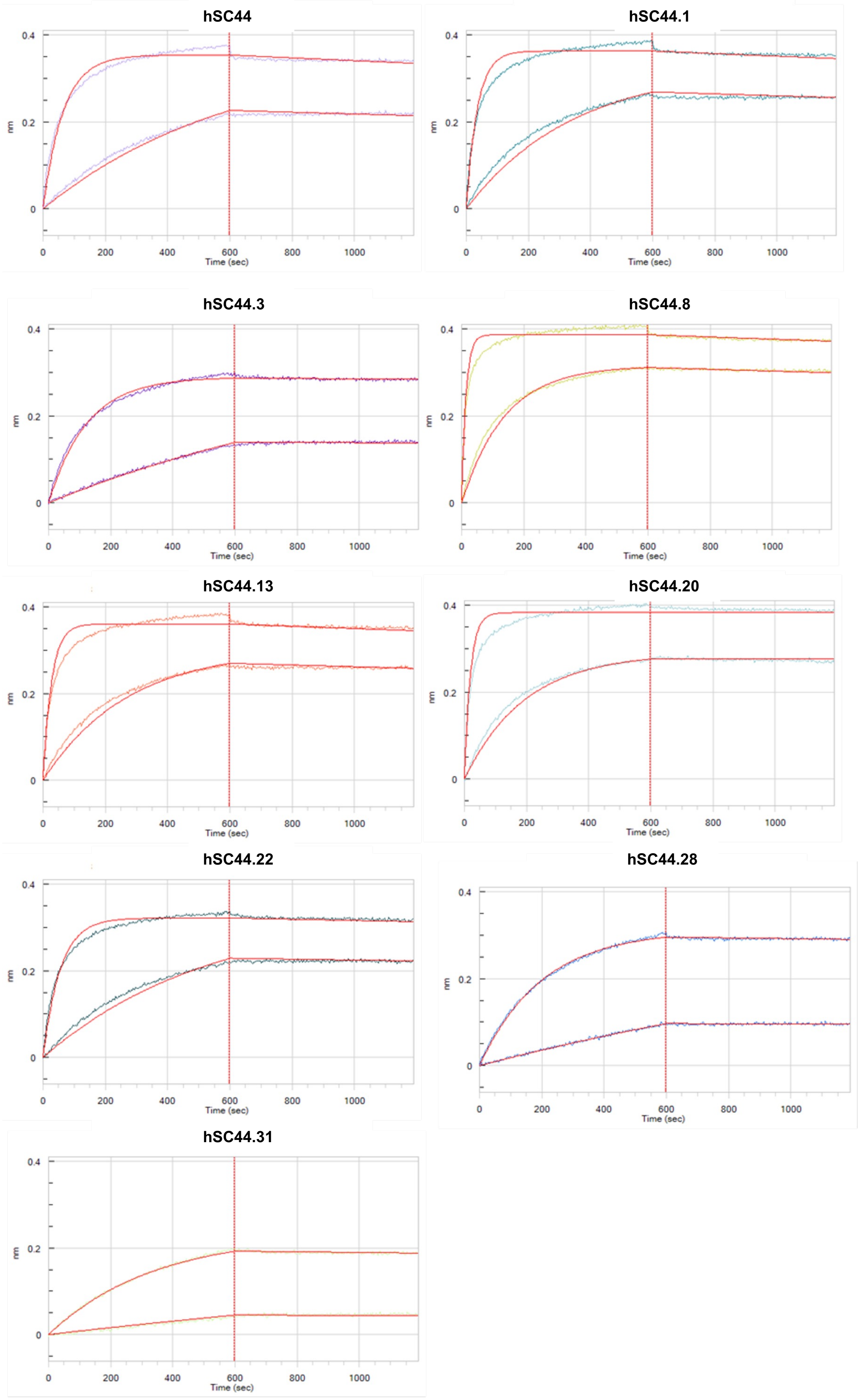
hSC44
hSC44.1
hSC44.3
hSC44.8
hSC44.13
hSC44.20
hSC44.22
hSC44.28
hSC44.31
nm
0.4
0.2
0
0
200
400
600
800
1000
Time (sec)

Fig. S7.

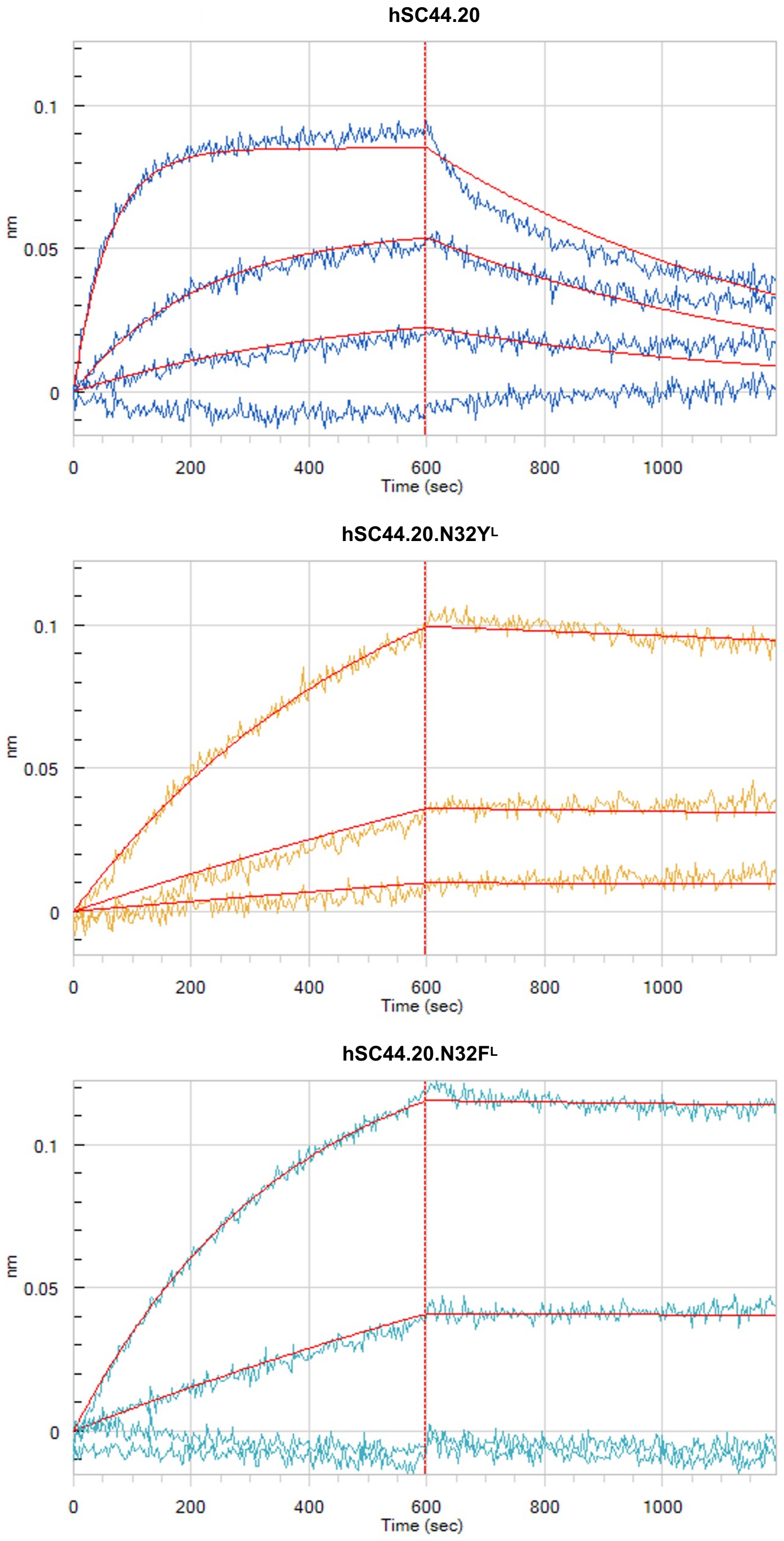
hSC44.20
nm
0.1
0.05
0
0
200
400
600
800
1000
Time (sec)
hSC44.20.N32Y^{L}
nm
0.1
0.05
0
0
200
400
600
800
1000
Time (sec)
hSC44.20.N32F^{L}
nm
0.1
0.05
0
0
200
400
600
800
1000
Time (sec)

Fig. S8.

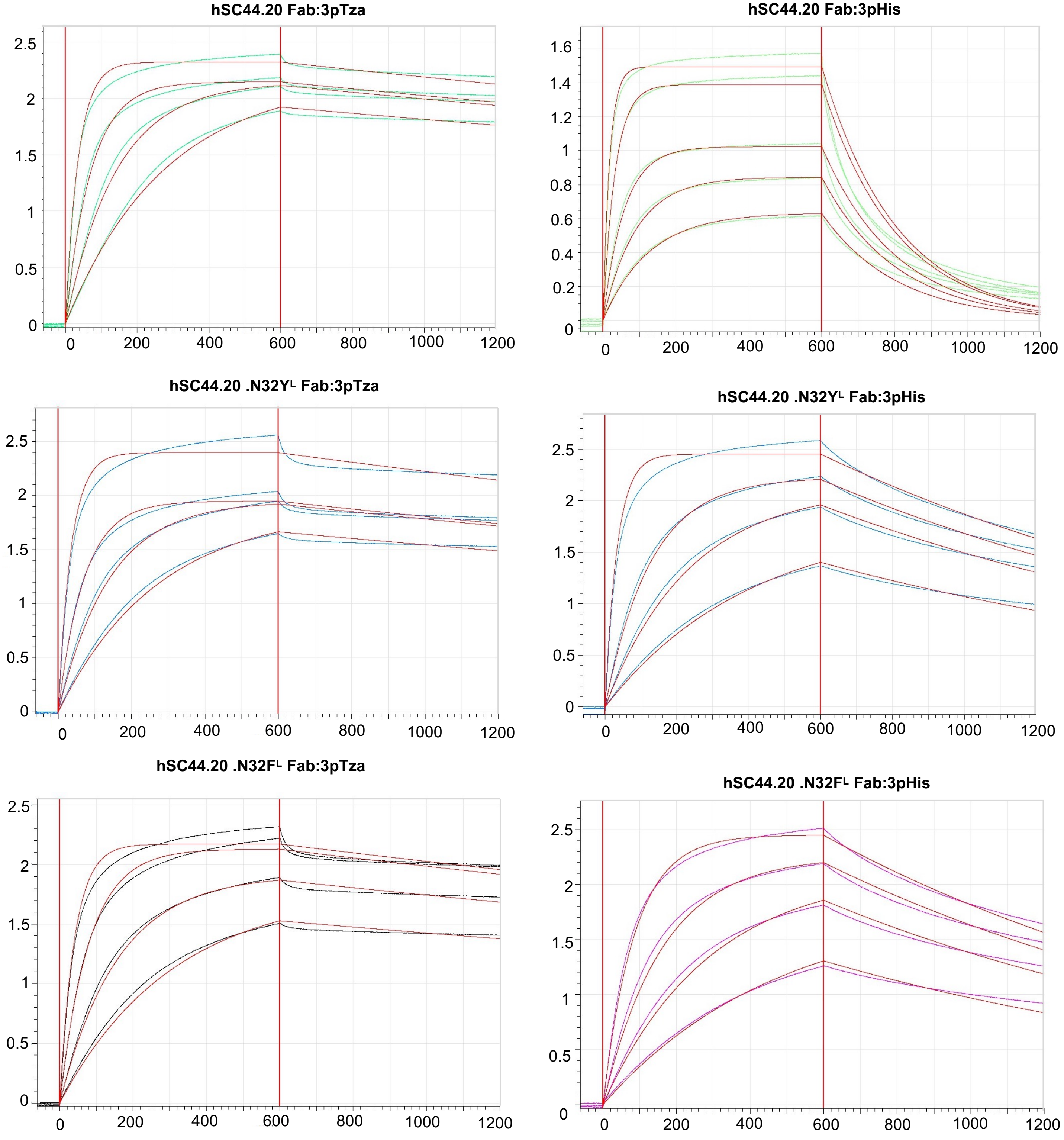
hSC44.20 Fab:3pTza
hSC44.20 Fab:3pHis
hSC44.20 .N32Y^{L} Fab:3pTza
hSC44.20 .N32Y^{L} Fab:3pHis
hSC44.20 .N32F^{L} Fab:3pTza
hSC44.20 .N32F^{L} Fab:3pHis

Fig. S9.

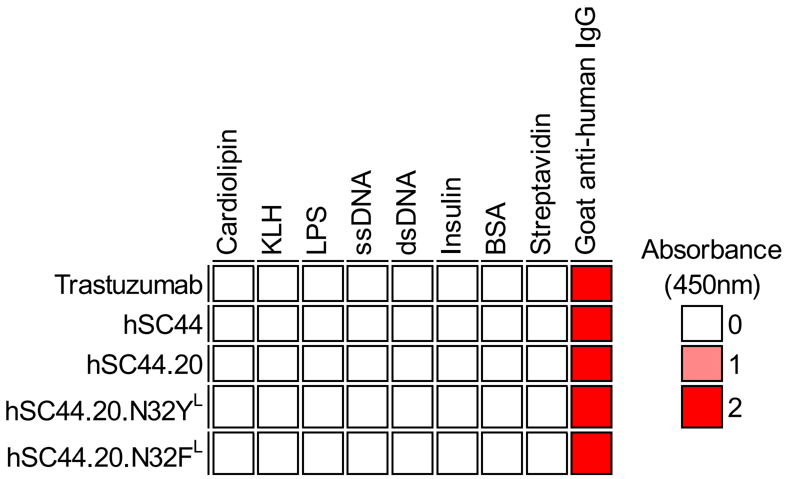

Cardiolipin
KLH
LPS
ssDNA
dsDNA
Insulin
BSA
Streptavidin
Goat anti-human IgG
Trastuzumab
hSC44
hSC44.20
hSC44.20.N32Y^L
hSC44.20.N32F^L
Absorbance
(450nm)
0
1
2

Fig. S10.

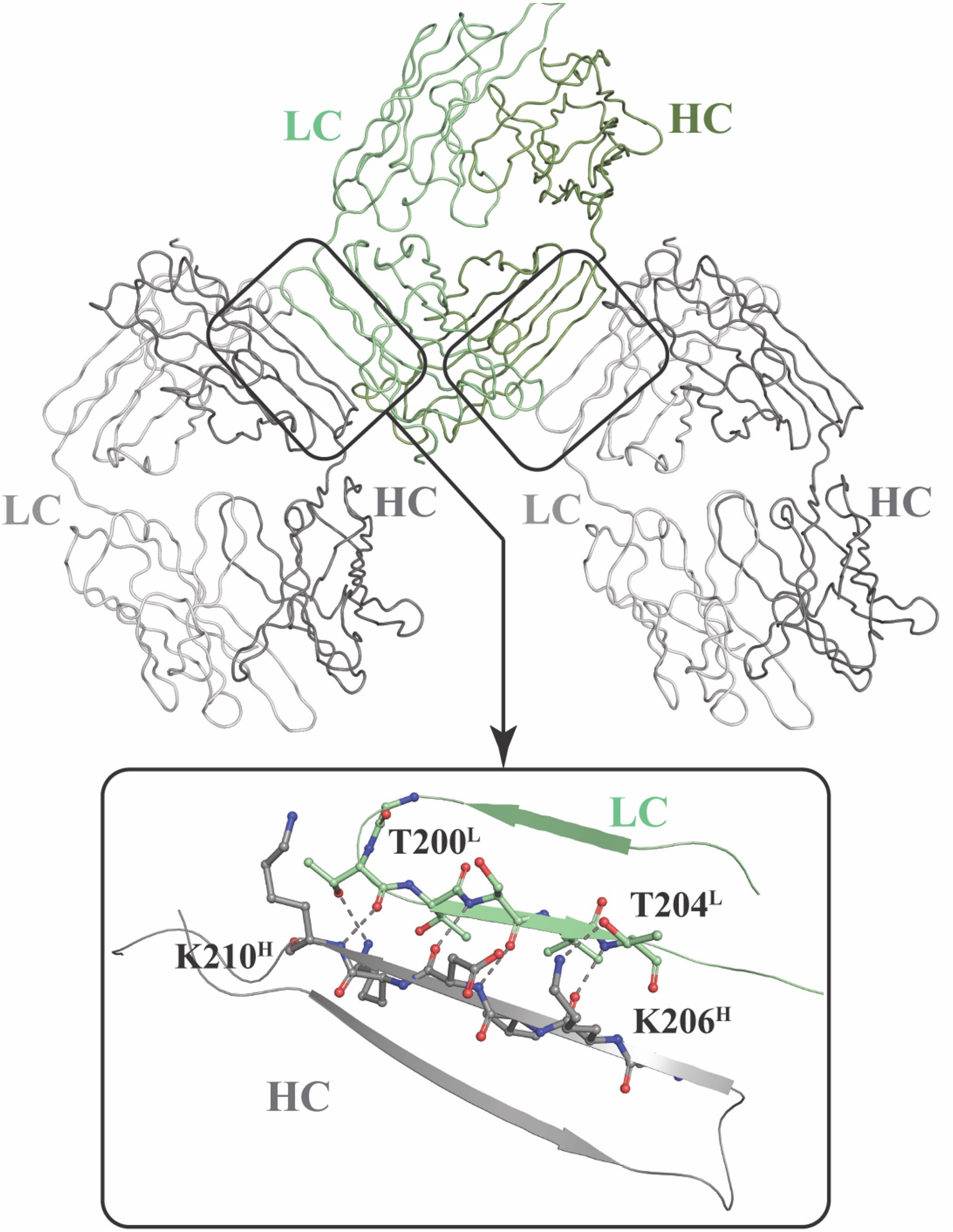

Fig. S11.

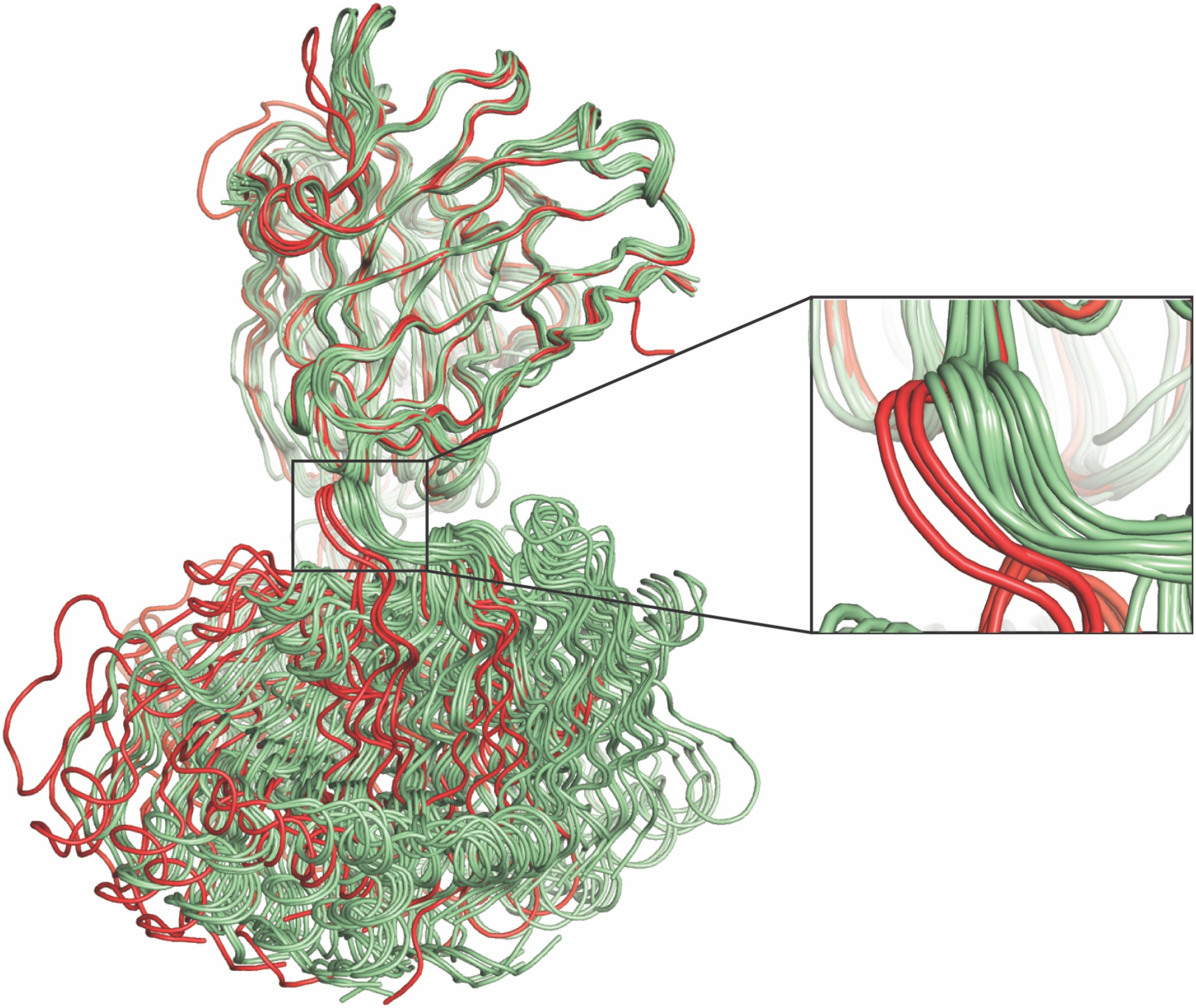

Fig. S12.

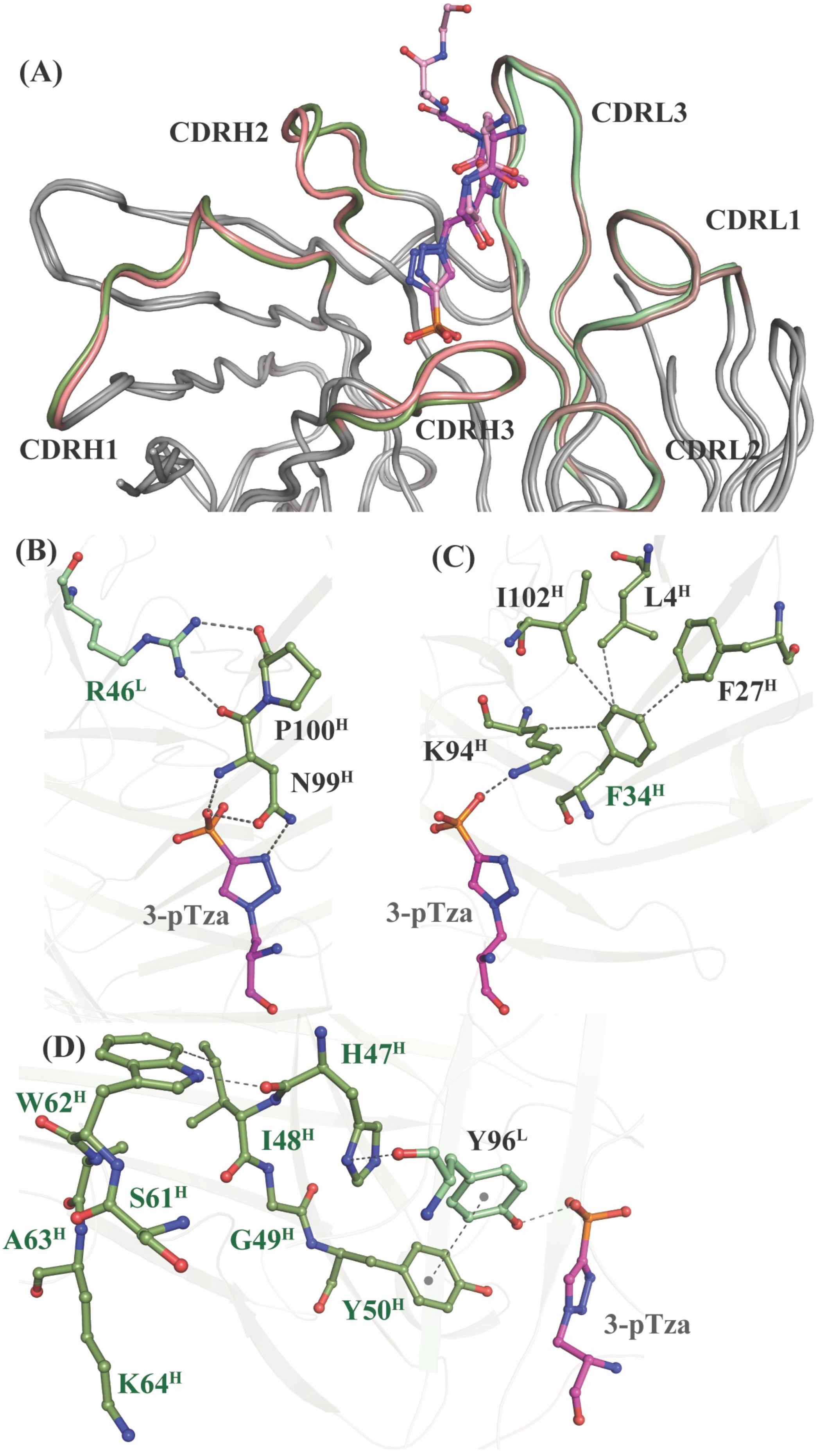

(A)
CDRH2
CDRL3
CDRL1
CDRH1
CDRH3
CDRL2
(B)
R46L
P100H
N99H
3-pTza
(C)
I102H
L4H
F27H
K94H
F34H
3-pTza
(D)
H47H
W62H
I48H
Y96L
S61H
A63H
G49H
Y50H
3-pTza
K64H

Fig. S13.

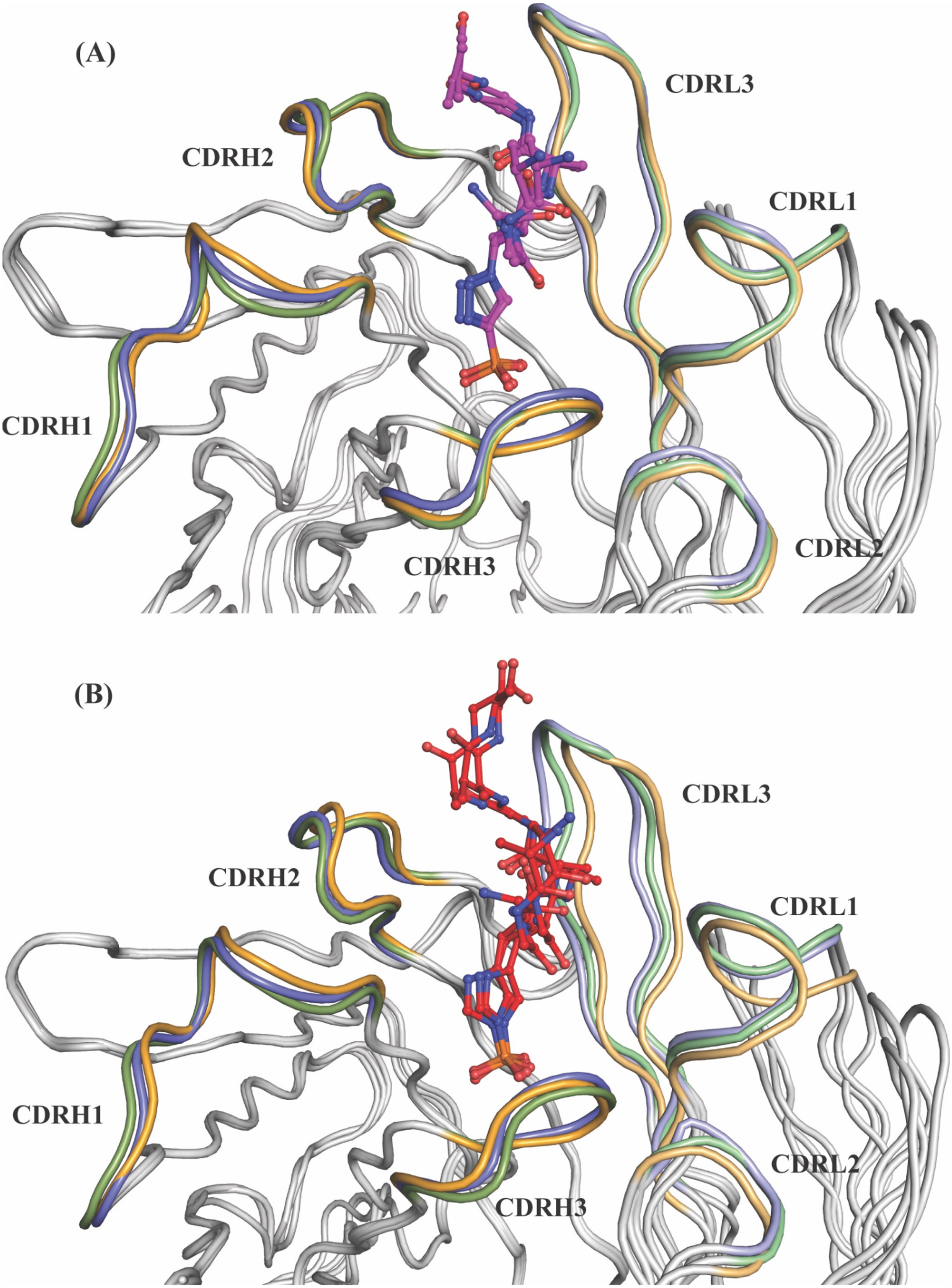
(A)
CDRL3
CDRH2
CDRL1
CDRH1
CDRL2
CDRH3
(B)
CDRL3
CDRH2
CDRL1
CDRH1
CDRL2
CDRH3

Fig. S14.

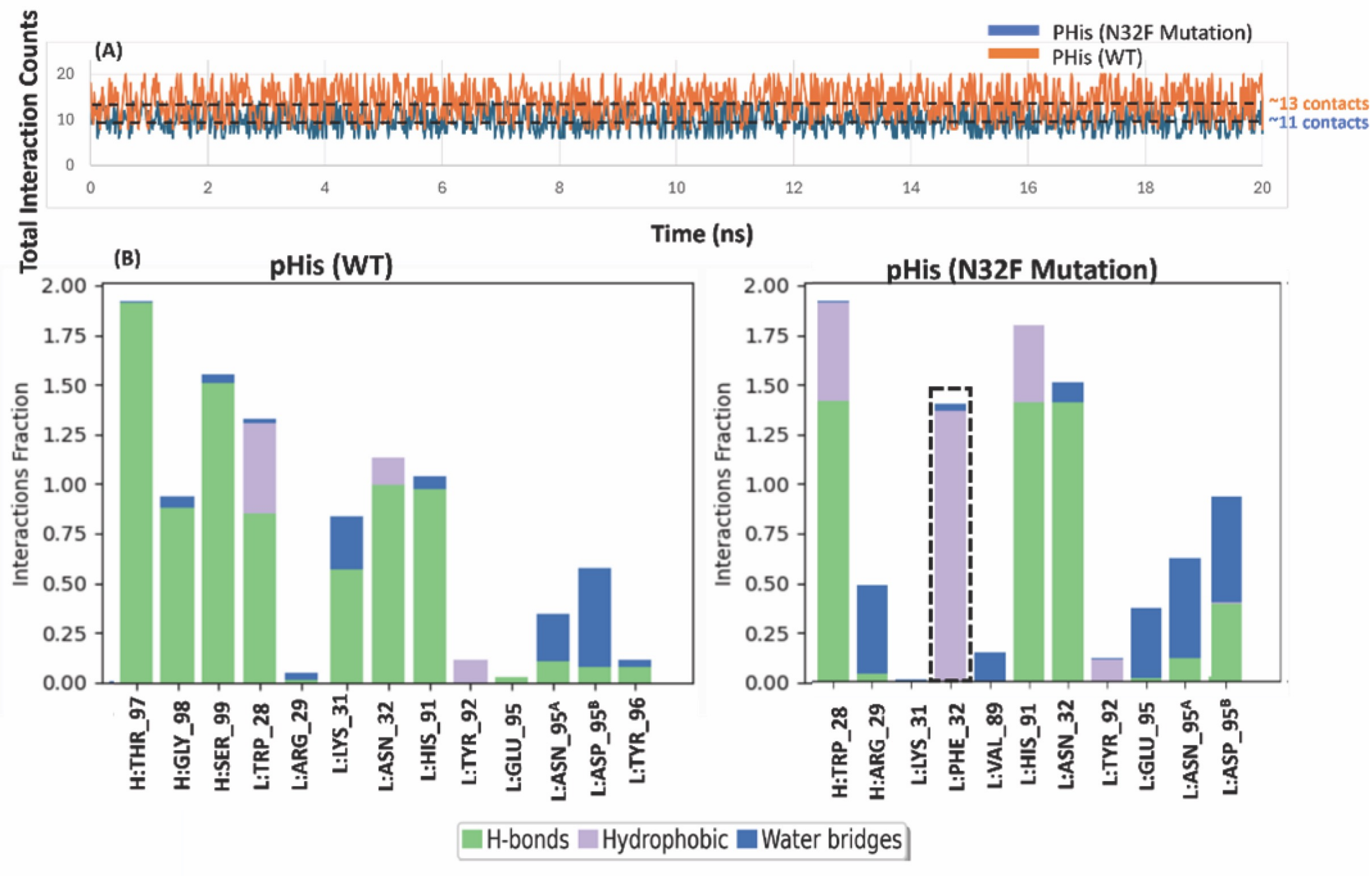
(A)
Total Interaction Counts
PHis (N32F Mutation)
PHis (WT)
~13 contacts
~11 contacts
Time (ns)
(B)
pHis (WT)
pHis (N32F Mutation)
Interactions Fraction
H:THR_97
H:GLY_98
H:SER_99
L:TRP_28
L:ARG_29
L:LYS_31
L:ASN_32
L:HIS_91
L:TYR_92
L:GLU_95
L:ASN_95A
L:ASP_95B
L:TYR_96
H:TRP_28
H:ARG_29
L:LYS_31
L:PHE_32
L:VAL_89
L:HIS_91
L:ASN_32
L:TYR_92
L:GLU_95
L:ASN_95A
L:ASP_95B
H-bonds
Hydrophobic
Water bridges

Fig. S15.

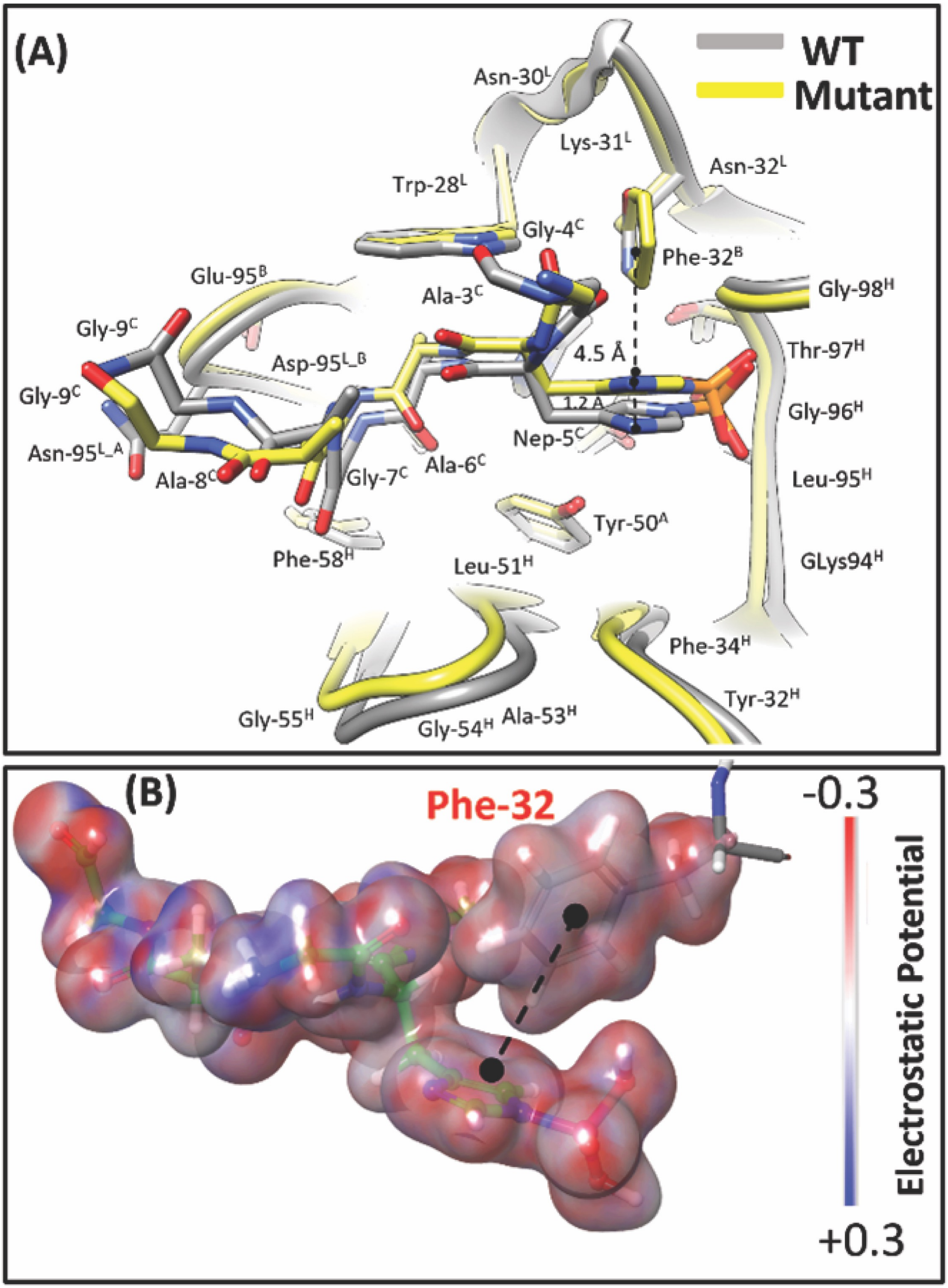
(A)
WT
Mutant
Asn-30L
Lys-31L
Asn-32L
Trp-28L
Gly-4C
Phe-32B
Glu-95B
Ala-3C
Gly-98H
Gly-9C
4.5 Å
Thr-97H
Asp-95L_B
Gly-9C
1.2 Å
Gly-96H
Nep-5C
Asn-95L_A
Ala-6C
Ala-8C
Gly-7C
Leu-95H
Tyr-50A
Phe-58H
Leu-51H
GLys94H
Phe-34H
Tyr-32H
Gly-55H
Gly-54H
Ala-53H
(B)
Phe-32
-0.3
Electrostatic Potential
+0.3

Fig. S16.

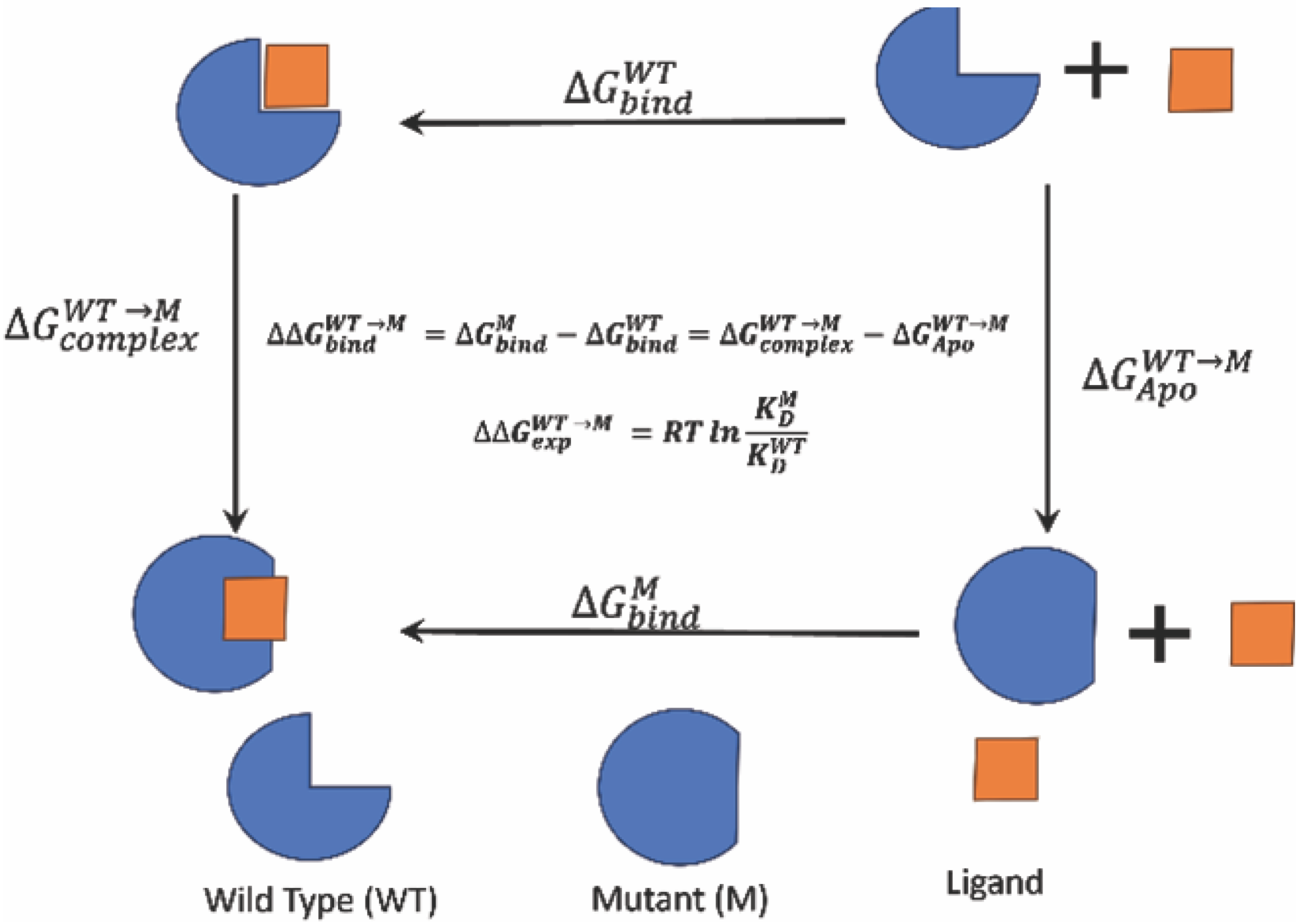

ΔG_{bind}^{WT}
$\Delta G_{complex}^{WT\rightarrow M}$
$\Delta\Delta G_{bind}^{WT\rightarrow M} = \Delta G_{bind}^{M} - \Delta G_{bind}^{WT} = \Delta G_{complex}^{WT\rightarrow M} - \Delta G_{Apo}^{WT\rightarrow M}$
$\Delta G_{Apo}^{WT\rightarrow M}$
$\Delta\Delta G_{exp}^{WT\rightarrow M} = RT\ ln\frac{K_D^M}{K_D^{WT}}$
ΔG_{bind}^{M}
Wild Type (WT)
Mutant (M)
Ligand

Fig. S17.

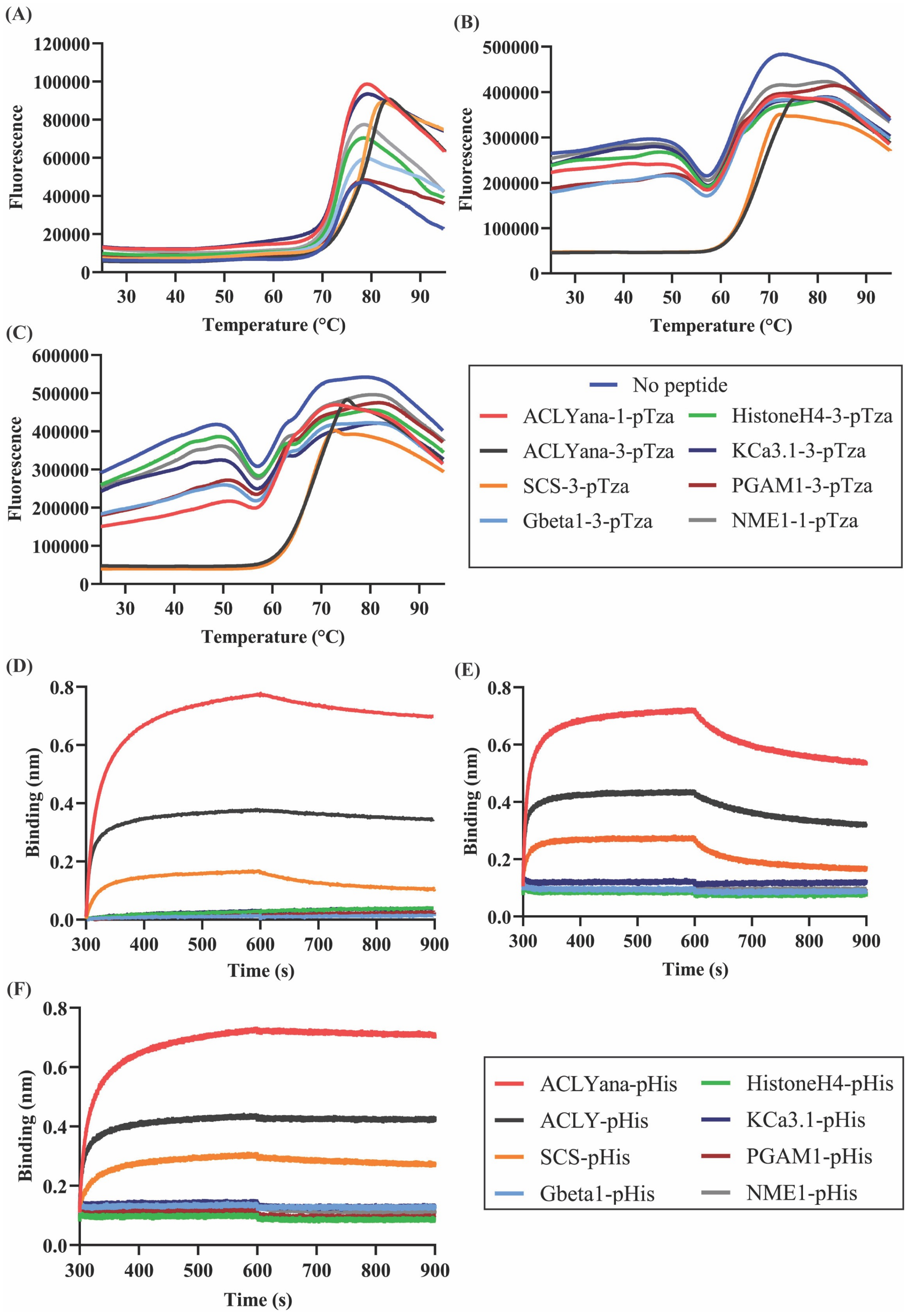
(A)
Fluorescence
Temperature (°C)
(B)
Fluorescence
Temperature (°C)
(C)
Fluorescence
Temperature (°C)
No peptide
ACLYana-1-pTza
ACLYana-3-pTza
SCS-3-pTza
Gbeta1-3-pTza
HistoneH4-3-pTza
KCa3.1-3-pTza
PGAM1-3-pTza
NME1-1-pTza
(D)
Binding (nm)
Time (s)
(E)
Binding (nm)
Time (s)
(F)
Binding (nm)
Time (s)
ACLYana-pHis
ACLY-pHis
SCS-pHis
Gbeta1-pHis
HistoneH4-pHis
KCa3.1-pHis
PGAM1-pHis
NME1-pHis

Fig. S18.

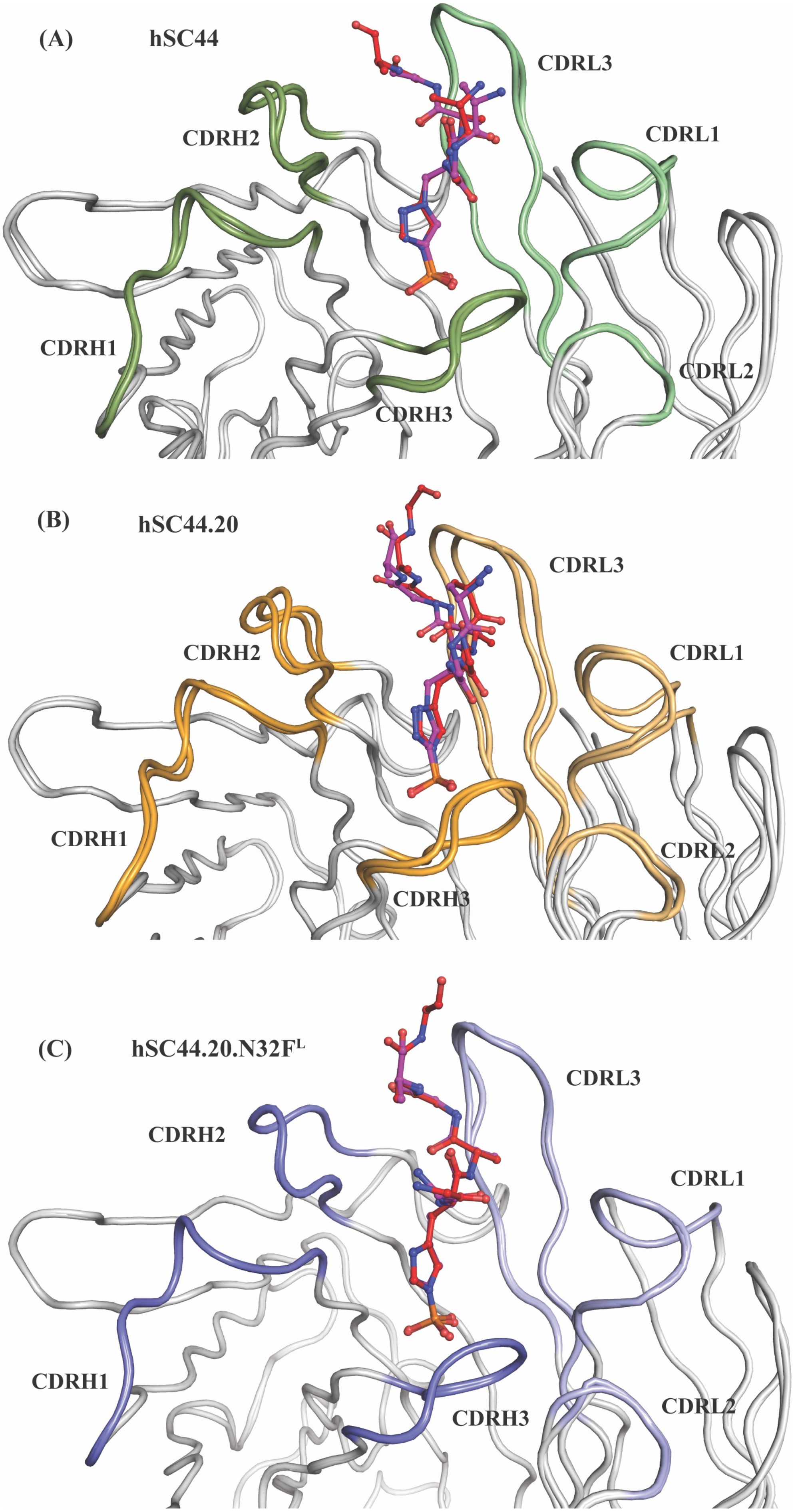

(A)
hSC44
CDRL3
CDRH2
CDRL1
CDRH1
CDRL2
CDRH3
(B)
hSC44.20
CDRL3
CDRH2
CDRL1
CDRH1
CDRL2
CDRH3
(C)
hSC44.20.N32F^L
CDRL3
CDRH2
CDRL1
CDRH1
CDRH3
CDRL2

Fig. S19.

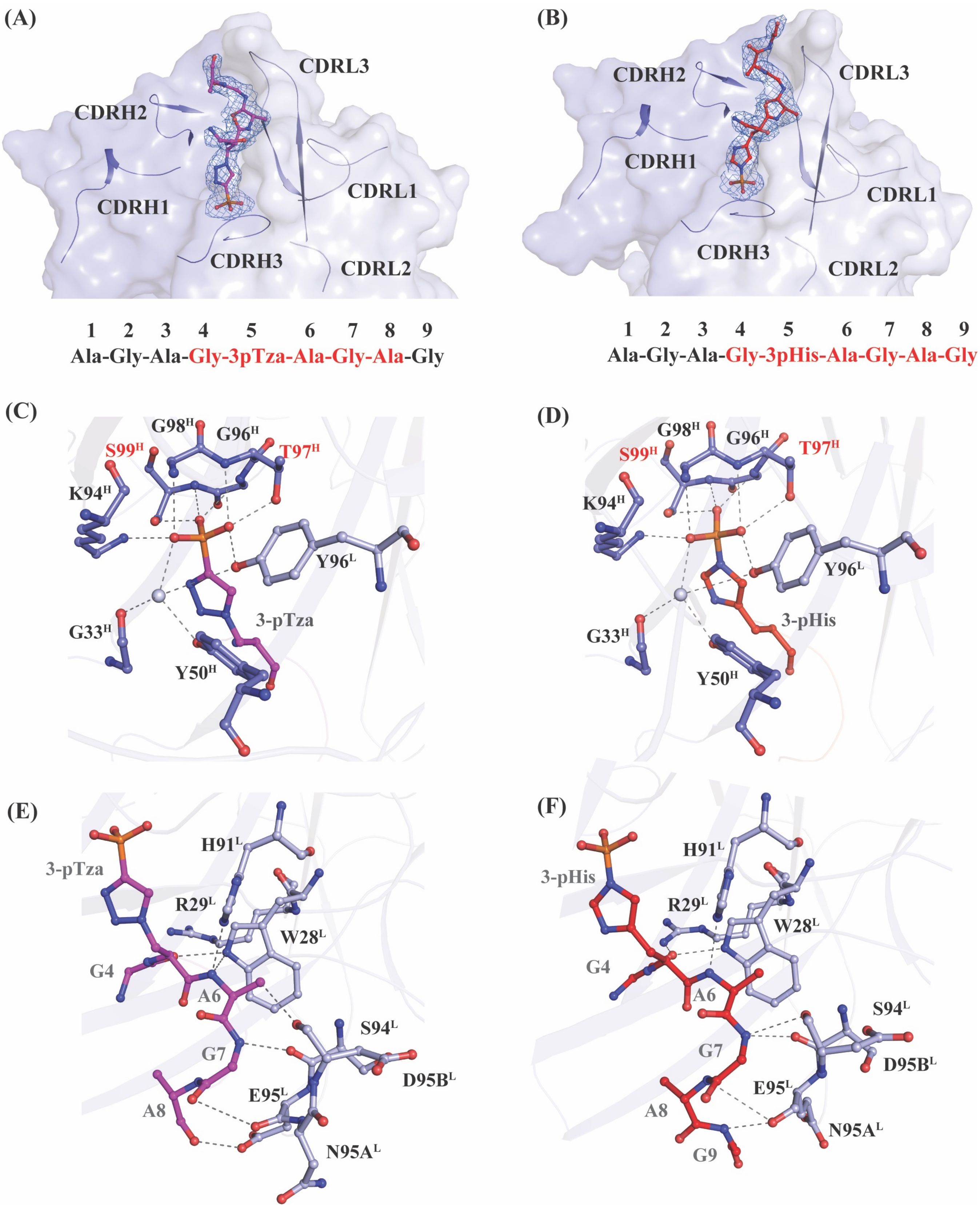
(A)
CDRL3
CDRH2
CDRH1
CDRL1
CDRH3
CDRL2
1 2 3 4 5 6 7 8 9
Ala-Gly-Ala-Gly-3pTza-Ala-Gly-Ala-Gly
(B)
CDRH2
CDRL3
CDRH1
CDRL1
CDRH3
CDRL2
1 2 3 4 5 6 7 8 9
Ala-Gly-Ala-Gly-3pHis-Ala-Gly-Ala-Gly
(C)
G98H
G96H
S99H
T97H
K94H
Y96L
3-pTza
G33H
Y50H
(D)
G98H
G96H
S99H
T97H
K94H
Y96L
3-pHis
G33H
Y50H
(E)
3-pTza
H91L
R29L
W28L
G4
A6
S94L
G7
D95BL
E95L
A8
N95AL
(F)
3-pHis
H91L
R29L
W28L
G4
A6
S94L
G7
D95BL
E95L
A8
N95AL
G9

Fig. S20.

(A)

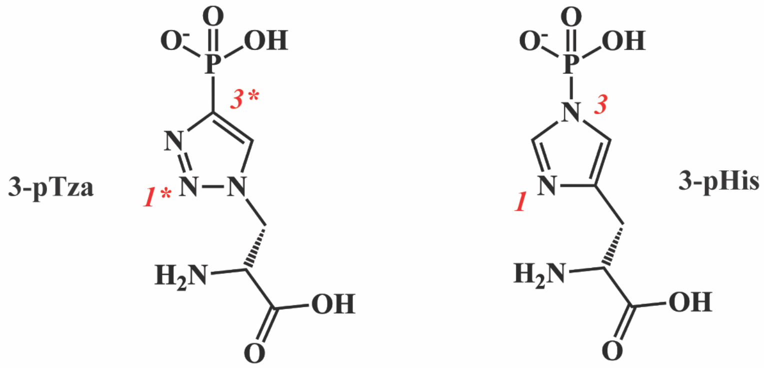
3-pTza
3-pHis
1*
3*
1
3
O
OH
P
N
H2N
O-

(B)

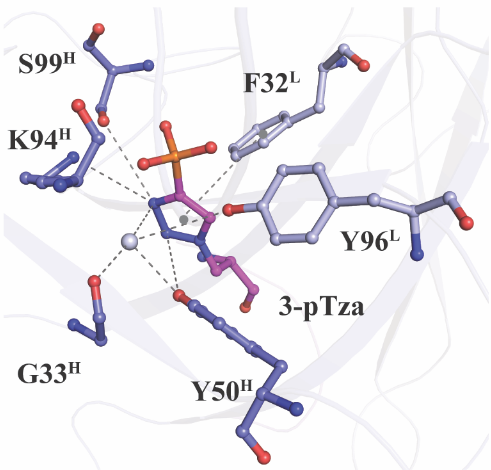
S99H
F32L
K94H
Y96L
3-pTza
G33H
Y50H

(C)

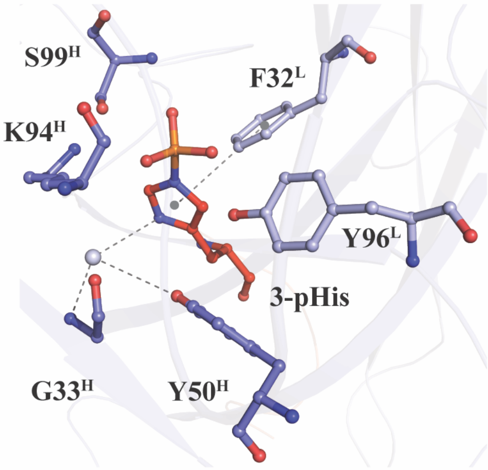
S99H
F32L
K94H
Y96L
3-pHis
G33H
Y50H

Fig. S21.

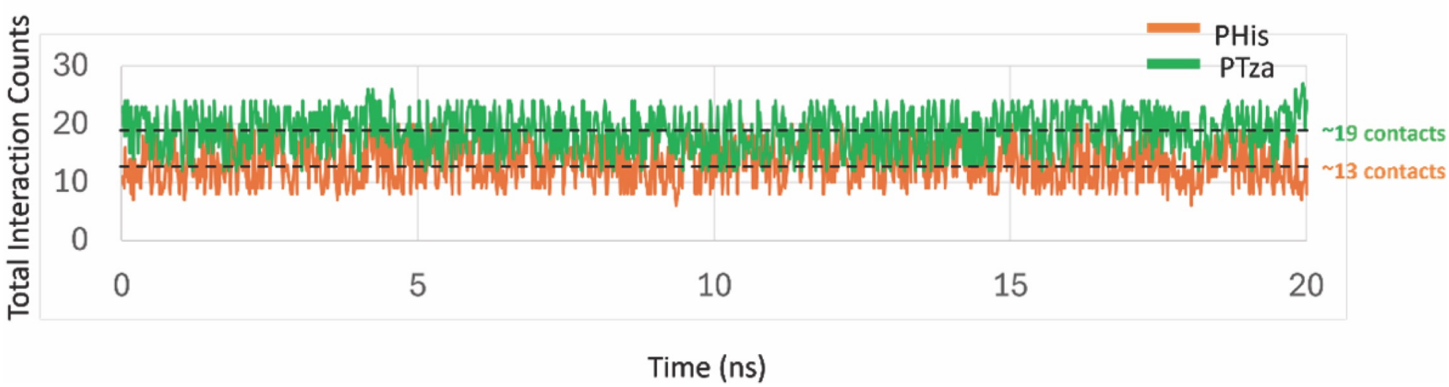

PHis
PTza
~19 contacts
~13 contacts
Total Interaction Counts
30
20
10
0
0
5
10
15
20
Time (ns)

Fig. S22.

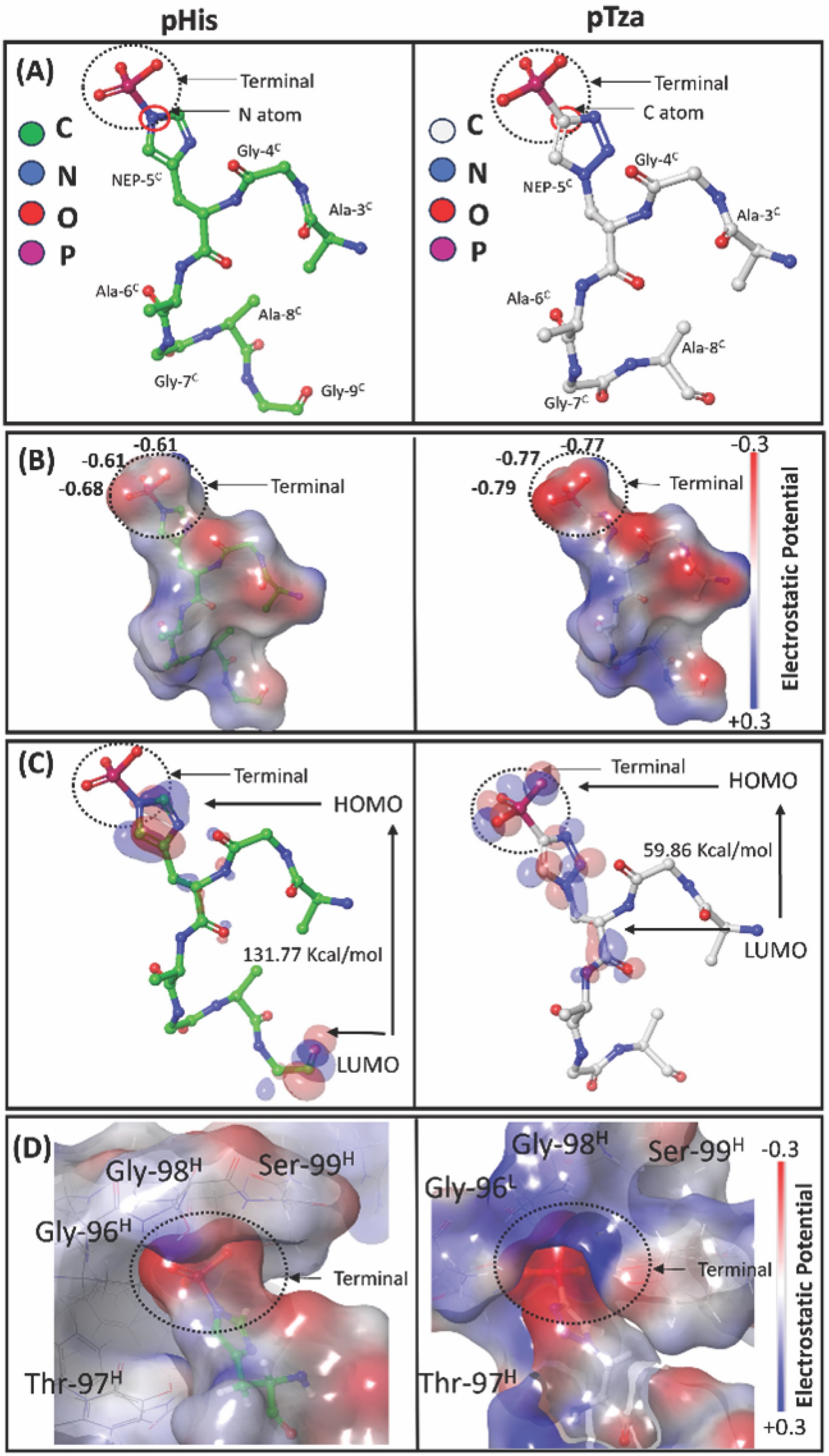

pHis
pTza
(A)
Terminal
N atom
C
N
O
P
Gly-4C
NEP-5C
Ala-3C
Ala-6C
Ala-8C
Gly-7C
Gly-9C
Terminal
C atom
C
N
O
P
Gly-4C
NEP-5C
Ala-3C
Ala-6C
Ala-8C
Gly-7C
(B)
-0.61
-0.61
-0.68
Terminal
-0.77
-0.77
-0.79
Terminal
-0.3
Electrostatic Potential
+0.3
(C)
Terminal
HOMO
131.77 Kcal/mol
LUMO
Terminal
HOMO
59.86 Kcal/mol
LUMO
(D)
Gly-98H
Ser-99H
Gly-96H
Terminal
Thr-97H
Gly-98H
Ser-99H
Gly-96L
Terminal
Thr-97H
-0.3
Electrostatic Potential
+0.3

Fig. S23.

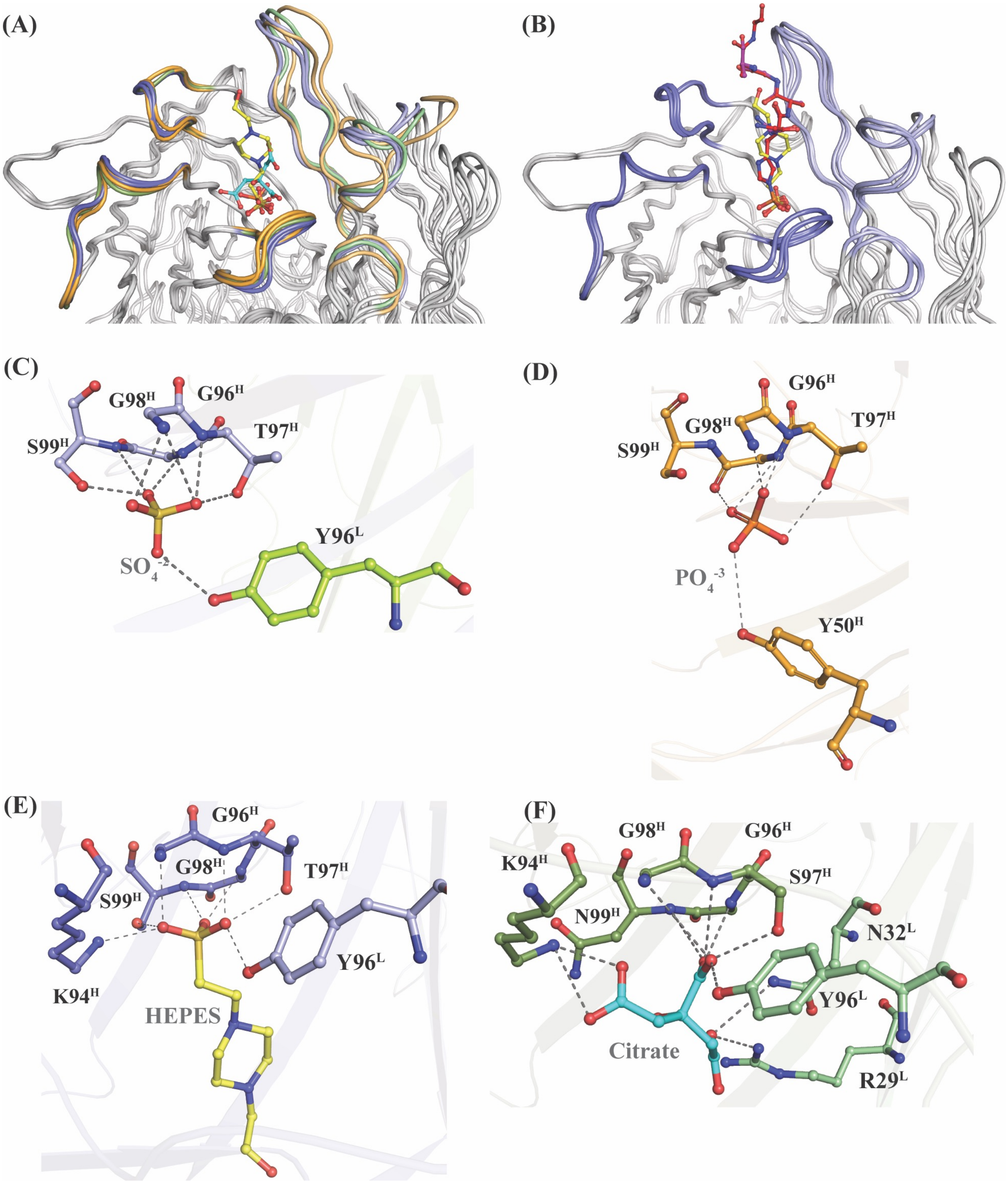
(A)
(B)
(C)
G98H
G96H
T97H
S99H
Y96L
SO4-2
(D)
G96H
G98H
T97H
S99H
PO4-3
Y50H
(E)
G96H
G98H
T97H
S99H
K94H
Y96L
HEPES
(F)
G98H
G96H
K94H
S97H
N99H
N32L
Y96L
Citrate
R29L

Fig. S24.

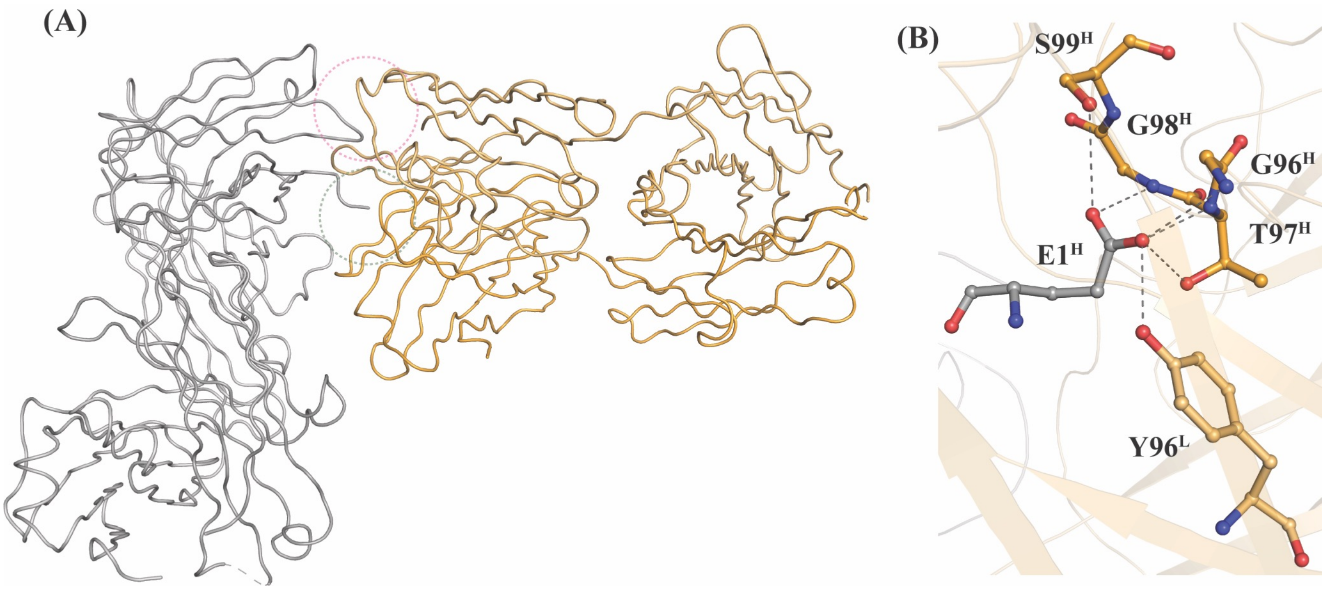

Table S1.

Table S1. Mutagenic oligonucleotides used to generate libraries hSC44 1-6.

Name	Mutagenic Oligonucleotide Sequence
hSC44.L1.1	CGTGCCAGTCAGTCCGTG(N4:10101070)(N3:10107010)(N3)(N2:10701010)(N3)(N4)AAC(N1:70101010)(N1)(N1)(N1)(N1)(N2)GTAGCCTGGTATCAACAG
hSC44.L1.2	CGTGCCAGTCAGTCCGTG(N4:10101070)(N3:10107010)(N3)CGTAACAAG(N1:70101010)(N1)(N2:10701010)GTAGCCTGGTATCAACAG
hSC44.L3.1	TACTGTGTGGGC(N2:10701010)(N1:70101010)(N4:10101070)TATGGCAGCGAAAACGATGCGTAT(N4)(N1)(N2)GCGTTCGGACAGGGTACC
hSC44.L3.2	TACTGTGTGGGC(N2:10701010)(N1:70101010)(N4:10101070)TAT(N3:10107010)(N3)(N2)(N1)(N3)(N2)(N3)(N1)(N1)(N1)(N1)(N2)(N3)(N1)(N4)(N3)(N2)(N3)TAT(N4)(N1)(N2)GCGTTCGGACAGGGTACC
hSC44.H1.1	GGCTTCAGCATTGATAGC(N4:10101070)(N1:70101010)(N4)(N3:10107010)(N3)(N2:10701010)TTTAGCTGGGTGCGTCAGGCC
hSC44.H2.1	CTGGAACATATTGGC(N4:10101070)(N1:70101010)(N4)CTG(N1)(N2:10701010)(N2)(N3:10107010)(N2)(N2)GGCGGCCGTGCGTTTTATGCC
hSC44.H2.2	CTGGAACATATTGGCTATCTG(N1:70101010)(N2:10701010)(N2)GCGGGCGGC(N2)(N3:10107010)(N4:10101070)GCGTTTTATGCCAGCTGG
hSC44.H3.1	GTCTATTATTGTGCT(N1:70101010)(N1)(N1)(N2:10701010)(N4:10101070)(N4)(N3:10107010)(N3)(N2)(N1)(N3)(N2)(N3)(N3)(N2)(N1)(N1)(N2)(N2)(N2)(N3)(N3)(N4)(N4)GCGATTTGGGGTCAAGGAACC
hSC44.H3.2	GTCTATTATTGTGCT(N1:70101010)(N1)(N3:10107010)(N2:10701010)(N4:10101070)(N4)(N3)(N3)(N2)(N1)(N3)(N2)GGC(N1)(N1)(N2)CCG(N3)(N4)(N4)GCGATTTGGGGTCAAGGAACC
hSC44.V.1	AGCATTGATAGCTATGGC(N4:10101070)(N4)(N4)(N1:70101010)(N3:10107010)(N2:10701010)TGGGTGCGTCAGGCCCCG
hSC44.V.2	GGTAAGGGCCTGGAA(N2:10701010)(N1:70101010)(N4:10101070)(N1)(N4)(N4)GGC(N4)(N1)(N4)CTGACCGCGGGC
hSC44.V.3	GGCCGTGCG(N4:10101070)(N4)(N4)TATGCC(N1:70101010)(N3:10107010)(N2:10701010)(N4)(N3)(N3)(N3)(N2)(N2)AAG(N1)(N3)(N2)CGT(N1)(N3)(N2)ACTATAACC(N2)(N3)(N4)AACACAAAC
hSC44.V.4	GAAAACACA(N3:10107010)(N4:10101070)(N4)ACCCTAAAAATGAACAGC

Table S2.

Table S2. Final concentrations of reagents used on various days of affinity selections.

	Day 1	Day 2	Day 3	Day 4	Day 5
BSA†	1%	1%	1%	1%	1%
Streptavidin†	10μg/mL	-	10μg/mL	-	10μg/mL
Neutravidin†	-	10μg/mL	-	10μg/mL	-
No PO_4^- Peptide*,†	-	10nM	25nM	50nM	100nM
1-pTza Peptide*,†	-	1nM	5nM	10nM	20nM
3-pTza/pHis Peptide*,‡	100nM	100nM	50nM	25nM	10nM
No. Wells	24	12	8	6	4
No. Washes	4	6	8	10	12

†Negative selections, ‡Positive Selections

*Captured using 2μg/mL streptavidin/neutravidin on alternating days

Table S3.

Table S3. Affinity measurements of hSC44 IgG variants against 3-pTza peptides using bio-layer interferometry.

	3-pTza Peptide		
**IgG**	**K_D (M)**	**K_a (1/Ms)**	**K_d (1/s)**
hSC44.1	5.88E-10	1.41E+05	8.32E-05
hSC44.3	3.52E-10	4.07E+04	1.43E-05
hSC44.8	1.85E-10	3.59E+05	6.66E-05
hSC44.13	4.05E-10	1.82E+05	7.37E-05
hSC44.20	<1.0E-12	2.57E+05	<1.0E-07
hSC44.20.N32F^L	1.54E-09	3.73E+04	5.73E-05
hSC44.20.N32Y^L	<1.0E-12	3.51E+04	<1.0E-07
hSC44.22	4.95E-10	9.06E+04	4.49E-05
hSC44.28	1.21E-09	2.49E+04	3.00E-05
hSC44.31	2.71E-09	1.47E+04	3.98E-05

Table S4.

Table S4. Affinity measurements of hSC44 IgG variants against 3-pHis peptides using bio-layer interferometry.

	3-pHis Peptide		
**IgG**	**K_D (M)**	**K_a (1/Ms)**	**K_d (1/s)**
hSC44.20	8.21E-07	7.55E+04	1.60E-03
hSC44.20.N32F^L	1.68E-09	1.39E+04	1.64E-05
hSC44.20.N32Y^L	9.50E-09	8.88E+03	2.61E-05

Table S5.

Table S5. Elbow angles of variants of hSC44 Fabs

Fab	Ligand	Protomers	Elbow angle (°)
hSC44.S1C	ACLYana-3-pTza peptide	HL	138.9
		AB	157.3
hSC44.S1C	ACLYana-3-pHis peptide	HL	137.8
		AB	157.1
hSC44.S1C	-	HL	138.9
		AB	159.7
hSC44.S1C.20	ACLYana-3-pTza peptide	HL	135.9
hSC44.S1C.20	ACLYana-3-pHis peptide	HL	135.8
hSC44.S1CE.20	-	HL	165.3
hSC44.S1CE.20	-	HL	172.6
hSC44.S1C.20.N32F^{L}	ACLYana-3-pTza peptide	HL	165.1
		AB	147.9
hSC44.S1C.20.N32F^{L}	ACLYana-3-pHis peptide	HL	165.4
		AB	149.0
hSC44.S1C.20.N32F^{L}	-	HL	156.2
hSC44.S1CE.20.N32F^{L}	-	HL	156.1

Table S6.

Table S6. Data collection and refinement statistics for newly solved Fab-Antigen crystal structures.

Table S6. X-ray data collection and refinement statistics for newly solved Fab-Antigen structures.

**Structure Name**	**hSC44.S1C**	**hSC44.S1C**	**hSC44.S1C**	**hSC44.S1C.20**	**hSC44.S1C.20**	**hSC44.S1CE.20**	**hSC44.S1CE.20**	**hSC44.S1C.20.N32F^{L}**	**hSC44.S1C.20. N32F^{L}**	**hSC44.S1C.20.N32F^{L}**	**hSC44.S1CE.20.N32F^{L}**
**Combining site ligand**	**AGAG-3pTza-AGAG**	**AGAG-3pHis-AGAG**	**No peptide (citrate)**	**AGAG-3pTza-AGAG**	**AGAG-3pHis-AGAG**	**No peptide (GluH1')**	**No peptide (PO4)**	**AGAG-3pTza-AGAG**	**AGAG-3pHis-AGAG**	**No peptide (SO4)**	**No peptide (HEPES)**
**Data collection**											
Beamline	SSRL 12-1	SSRL 12-1	APS 23-ID-B	APS 23-ID-D	SSRL 12-1	ALS 5.0.1	ALS 5.0.1	ALS 5.0.1	ALS 5.0.1	ALS 5.0.1	ALS 5.0.1
Wavelength (Å)	0.97946	0.97946	1.03317	1.0332	0.97946	0.97741	0.97741	0.97741	0.97741	0.97741	0.97741
Resolution (Å)[a]	39.34-1.95	38.72-2.20	46.91-1.75	41.04-1.85	34.82-1.90	47.17-2.45	49.79-2.40	47.25-1.98	47.19-2.09	48.05-1.94	46.27-1.84
Space group	$P2_12_12$	$P2_12_12$	$P2_12_12$	C2	$P2_1$	$P2_12_12_1$	$P4_32_12$	$P2_12_12_1$	$P2_12_12_1$	$P2_12_12_1$	$P2_12_12_1$
Unit cell (Å)	92.50, 139.62, 73.62	93.25, 139.06, 73.71	93.82, 138.85, 73.21	79.96, 73.12, 88.07	44.11, 72.61, 70.33	49.7, 73.3, 148.5	72.3, 72.3, 205.9	71.55, 73.95, 239.65	71.28, 74.03, 239.28	69.47, 73.25, 96.11	48.57, 72.07, 152.2
(°)	90, 90, 90	90, 90, 90	90, 90, 90	90, 111.24, 90	90, 98.21, 90	90, 90, 90	90, 90, 90	90, 90, 90	90, 90, 90	90, 90, 90	90, 90, 90
Total reflections	733,426	637,037	688,286	155,586	148,666	127,101	219,439	538,821	418,880	284,579	293,288
Unique reflections	68,051(3151)	46,924(2432)	95,210(4089)	40,163(1984)	33,299(1333)	19,822(839)	22,383(1088)	89,758(4417)	71,747(3417)	36,687(1806)	46,607(2191)
Multiplicity	10.8(7.8)	12.9(9.4)	7.2(5.5)	3.9(3.3)	4.5(3.6)	6.4(5.7)	9.8(6.2)	6.0(5.6)	5.8(5.2)	7.8(7.6)	6.3(4.5)
Completeness (%)	97.5(91.3)	99.9(99.9)	98.1(85.3)	99.8(99.4)	96.1(77.1)	96.3(84.2)	100(99.7)	100(100)	95.2(92.7)	100(100)	99.2(94.4)
Mean (I)/ σ_I	13.1(1.7)	11.8(1.8)	10.0(1.6)	6.9(1.1)	9.9(2.8)	19.1(2.1)	28.0(2.7)	8.9(1.5)	13.9(1.5)	11.4(1.0)	16.0(1.2)
R_{merge}[b](%)	14.3(193)	22.1(483)	12.4(117)	13.1(128)	16.2(120)	6.3(85.4)	7.1(76.1)	12.5(135)	10.1(130)	18.0(238)	10.0(178)
R_{meas}[c](%)	14.9(205)	23.0(512)	13.3(129)	15.1(151)	18.3(141)	6.9(94.1)	7.5(83.0)	13.7(150)	11.0(144)	19.3(254)	10.8(200)
R_{pim}[d](%)	4.4(67.3)	6.4(166)	4.9(52.9)	7.4(79.2)	8.2(71.9)	2.7(38.7)	2.3(32.2)	5.6(63.6)	4.4(61.1)	6.7(88.4)	4.2(90.4)
$CC_{1/2}$[e](%)	99.8(47.2)	99.8(56.5)	99.7(41.1)	99.5(44.1)	99.2(55.0)	99.9(70.6)	99.9(80.2)	99.5(39.6)	99.7(43.2)	99.3(37.4)	99.6(37.1)
**Refinement**											
Refinement resolution (Å)[a]	39.34-1.95	38.72-2.20	46.91-1.75	41.04-1.85	34.82-1.90	47.17-2.45	49.79-2.40	47.25-1.98	47.2-2.09	48.05-1.94	46.27-1.84
# reflections in refinement (work/free)	64,623/3393	46,924/2489	90,339/4802	38,147/1978	31,562/1721	18,793/985	21,221/1079	85,368/4291	68,152/3509	34,775/1846	44,224/2299
R_{work}/R_{free} (%)	21.2/26.0	23.0/27.5	21.0/23.9	20.5/24.3	17.2/21.3	24.4/29.0	25.8/29.3	22.4/25.7	21.8/24.5	21.8/24.7	20.4/23.6
# atoms (Fab/Peptide/Solvent)	6556/64/223	6556/69/64	6532/na/476	6514/57/283	6526/69/188	3286/na/10	3287/na/34	6589/64/206	6552/72/334	3278/na/149	3336/na/480
RMS (bonds)	0.006	0.002	0.006	0.003	0.005	0.005	0.003	0.008	0.002	0.003	0.005
RMS (angles)	0.91	0.6	0.83	0.75	0.77	0.71	0.61	0.94	0.52	0.66	0.86
Ramachandran favoured/allowed/ outliers (%)	96.9/2.9/0.2	97.6/2.4/0	96.7/2.9/0.4	97.7/2.3/0	97.7/2.1/0.2	95.1/4.7/0.2	96.0/4.0/0	96.4/3.6/0	97.3/2.6/0.1	97.7/2.3/0	98.4/1.6/0
Ramachandran plot Z score	0.02	-0.1	0.2	-0.1	0.8	-1.4	-1.6	-0.29	-0.81	0.06	0.39
Clashscore[f]	3.1	1.8	3.7	0.6	1.5	3.2	4.6	2.1	1.4	2.6	3.5
Wilson B ($Å^2$)	30	41	22	22	21	53	51	32	36	27	25
Average B ($Å^2$) for all atoms/Fab/ Peptide/Solvent	40/38/54/34	56/56/85/43	34/34/na/30	29/29/38/32	25/25/22/27	63/63/na/46	72/72/na/51	42/42/56/36	43/43/56/41	30/30/46/29	31/30/20/36
PDB ID	**8UJI**	**8UIT**	**8UIO**	**8UIH**	**8UIG**	**8UHT**	**8UHS**	**8UHP**	**8UHN**	**8UHJ**	**8UHH**

[a]Numbers in parentheses are for highest resolution shell

[b]$R_{merge} = \Sigma_{hkl} \Sigma_{i=1,n}|I_i(hkl) - <I(hkl)>| / \Sigma_{hkl} \Sigma_{i=1,n} I_i(hkl)$

[c]$R_{meas} = \Sigma_{hkl} \sqrt{(n/n-1)} \Sigma_{i=1,n}|I_i(hkl) - <I(hkl)>| / \Sigma_{hkl} \Sigma_{i=1,n} I_i(hkl)$

[d]$R_{pim} = \Sigma_{hkl} \sqrt{(1/n-1)} \Sigma_{i=1,n}|I_i(hkl) - <I(hkl)>| / \Sigma_{hkl} \Sigma_{i=1,n} I_i(hkl)$

[e]$CC_{1/2}$ = Pearson Correlation Coefficient between two random half datasets

[f]Number of unfavorable all-atom steric overlaps ≥ 0.4Å per 1000 atoms

Table S7.

Table S7. Constructs with ligand, crystallization conditions and cryoprotectant used for crystallization experiments

pHis Fab	Ligand	Crystallization condition	Cryoprotectant
hSC44.S1C (C1)	AGAG-3-pTza-AGAG	0.2 M Lithium citrate, 20% PEG3350	30 % Ethylene glycol
hSC44.S1C (A7)	AGAG-3-pHis-AGAG	0.2 M Calcium chloride, 20% PEG3350	30 % Ethylene glycol
hSC44.S1C (A2)	No ligand	0.2 M Lithium citrate, 20% PEG3350	30 % Ethylene glycol
hSC44.S1C.20 (J13)	AGAG-3-pTza-AGAG	0.2 M tri-potassium citrate, 20% PEG3350	25% PEG400
hSC44.S1C.20 (B5)	AGAG-3-pHis-AGAG	0.2 M tri-potassium citrate, 20% PEG3350	25% PEG400
hSC44.S1CE.20 (C16)	No ligand	0.1 M Tris pH 8.5, 8% PEG8000	30 % Ethylene glycol
hSC44.S1CE.20 (A10)	No ligand	0.1 M HEPES pH 7.5, 20% PEG4000, 10% 2-propanol	30 % Ethylene glycol
hSC44.S1C.20.N32F^L (E12)	AGAG-3-pTza-AGAG	0.08 M Sodium Cacodylate pH 6.5, 0.16 M Calcium acetate, 20% glycerol, 14.4% PEG8000	10% glycerol
hSC44.S1C.20.N32F^L (E14)	AGAG-3-pHis-AGAG	0.08 M Sodium Cacodylate pH 6.5, 0.16 M Calcium acetate, 20% glycerol, 14.4% PEG8000	10% glycerol
hSC44.S1C.20.N32F^L (C8)	No ligand	0.1 M Tris pH 8.5, 0.2 M Lithium sulfate, 40% PEG400	Well solution
hSC44.S1CE.20.N32F^L (C15)	No ligand	0.1 M HEPES pH 7.5, 20% PEG4000, 10% 2-propanol	30 % Ethylene glycol

Table S8.

Table S8: Changes in the ligand binding free energies caused by the N32F^L mutation. The first column shows the binding free energy differences from FEP calculations ($\Delta\Delta \boldsymbol{G}_{\boldsymbol{bind}}^{\boldsymbol{WT} \rightarrow \boldsymbol{M}}$) of 3-pHis, whereas the last column shows the experimentally obtained binding free energy differences.

System	Relative Binding Free energy<br>$\Delta\Delta G_{bind}$ (Kcal/mol)	K_D(M)	Experimental binding free energy $\Delta\Delta G_{bind}$ (Kcal/mol)
pHis_SC44H.20.F (N32F^L)	-1.1±0.4	1.68E-09	$RT \ln \frac{K_D^M}{K_D^{WT}}$ =-1.4
pHis_SC44H.20.F (WT)		2.10E-08	
