# Supplementary material for: Using phage display for rational engineering of a higher-affinity humanized 3’ phosphohistidine-specific antibody
Source: Commun Chem. 2025 Nov 27;8:381. doi: 10.1038/s42004-025-01768-9 (PMC12660843; doi:10.1038/s42004-025-01768-9)
Supplement: Supplementary file 2 — reporting summary [file 42004_2025_1768_MOESM2_ESM.pdf]

nature portfolio

Corresponding author(s): Tony Hunter
Last updated by author(s): Jan 15, 2025

Reporting Summary

Nature Portfolio wishes to improve the reproducibility of the work that we publish. This form provides structure for consistency and transparency in reporting. For further information on Nature Portfolio policies, see our Editorial Policies and the Editorial Policy Checklist.

Statistics

For all statistical analyses, confirm that the following items are present in the figure legend, table legend, main text, or Methods section.

n/a	Confirmed	
☐	☒	The exact sample size (*n*) for each experimental group/condition, given as a discrete number and unit of measurement
☐	☒	A statement on whether measurements were taken from distinct samples or whether the same sample was measured repeatedly
☒	☐	The statistical test(s) used AND whether they are one- or two-sided<br>*Only common tests should be described solely by name; describe more complex techniques in the Methods section.*
☒	☐	A description of all covariates tested
☒	☐	A description of any assumptions or corrections, such as tests of normality and adjustment for multiple comparisons
☒	☐	A full description of the statistical parameters including central tendency (e.g. means) or other basic estimates (e.g. regression coefficient) AND variation (e.g. standard deviation) or associated estimates of uncertainty (e.g. confidence intervals)
☒	☐	For null hypothesis testing, the test statistic (e.g. *F*, *t*, *r*) with confidence intervals, effect sizes, degrees of freedom and *P* value noted<br>*Give P values as exact values whenever suitable.*
☒	☐	For Bayesian analysis, information on the choice of priors and Markov chain Monte Carlo settings
☒	☐	For hierarchical and complex designs, identification of the appropriate level for tests and full reporting of outcomes
☒	☐	Estimates of effect sizes (e.g. Cohen's *d*, Pearson's *r*), indicating how they were calculated

Our web collection on statistics for biologists contains articles on many of the points above.

Software and code

Policy information about availability of computer code

Data collection	Data collection information is listed in relevant areas of the methods and acknowledgments section of this manuscript. The X-ray crystallography data were collected on APS beamline 23-ID-D, SSRL beamline 12-2 and ALS beamline 5.0.3 facilities.
Data analysis	ELISA data were analyzed using Prism 6 software (GraphPad Software Inc). Structural alignments were processed and visualized in PyMol (v.2.5.2). Library design and DNA sequencing was analyzed using Geneious v.10.0.09 (https://www.geneious.com). Biolayer interferometry data were analyzed using Octet Systems Software v.9.0 (FortèBio).The X-ray crystallography data were processed using HKL2000 and XDS programs and Phenix and CCP4 suites. Quantum Mechanics/Molecular Mechanics/Molecular Dynamics and Binding Affinity Simulations (FEP) was analyzed using Schrodinger 23-2 (https://www.schrodinger.com/life-science/download/release-notes/release-2023-2/), visualization was done using UCSF Chimera and Maestro-2023 from the Schrodinger suite tool.

For manuscripts utilizing custom algorithms or software that are central to the research but not yet described in published literature, software must be made available to editors and reviewers. We strongly encourage code deposition in a community repository (e.g. GitHub). See the Nature Portfolio guidelines for submitting code & software for further information.

Data

Policy information about availability of data

All manuscripts must include a data availability statement. This statement should provide the following information, where applicable:

- Accession codes, unique identifiers, or web links for publicly available datasets
- A description of any restrictions on data availability
- For clinical datasets or third party data, please ensure that the statement adheres to our policy

The data and results supporting this study are available within this manuscript and supplementary data files. The X-ray crystallography data (coordinates and structure factors) of hSC44 and engineered variants are deposited in the Protein Data Bank (www.rcsb.org) with the following accession codes- 8UJI (hSC44.S1C:AGAG-3pTza-AGAG), 8UIT (hSC44.S1C:AGAG-3pHis-AGAG), 8UIO (hSC44.S1C), 8UIH (hSC44.S1C.20:AGAG-3pTza-AGAG), 8UIG (hSC44.S1C.20:AGAG-3pHis-AGAG), 8UHT (hSC44.S1CE.20), 8UHS (hSC44.S1CE.20), 8UHP (hSC44.S1C.20.N32FL:AGAG-3pTza-AGAG), 8UHN (hSC44.S1C.20.N32FL:AGAG-3pHis-AGAG), 8UHJ (hSC44.S1C.20.N32FL) and 8UHH (hSC44.S1CE.20.N32FL). Additional data or materials used in this study, such as plasmids, for protein expression are available upon reasonable request.

Research involving human participants, their data, or biological material

Policy information about studies with human participants or human data. See also policy information about sex, gender (identity/presentation), and sexual orientation and race, ethnicity and racism.

Reporting on sex and gender	N/A
Reporting on race, ethnicity, or other socially relevant groupings	N/A
Population characteristics	N/A
Recruitment	N/A
Ethics oversight	N/A

Note that full information on the approval of the study protocol must also be provided in the manuscript.

Field-specific reporting

Please select the one below that is the best fit for your research. If you are not sure, read the appropriate sections before making your selection.

[x] Life sciences [ ] Behavioural & social sciences [ ] Ecological, evolutionary & environmental sciences

For a reference copy of the document with all sections, see nature.com/documents/nr-reporting-summary-flat.pdf

Life sciences study design

All studies must disclose on these points even when the disclosure is negative.

Sample size	Sample size and rationale behind each decision is described in the manuscript.
Data exclusions	Data acquisition and analysis (including any relevant filtering) with regards to structure determination is described in the methods section of this manuscript.
Replication	Replication for binding experiments to estimate affinity of engineered antibodies using ELISA or BLI is described in the methods section of this manuscript.
Randomization	Not relevant to our study.
Blinding	Not relevant to our study.

Reporting for specific materials, systems and methods

We require information from authors about some types of materials, experimental systems and methods used in many studies. Here, indicate whether each material, system or method listed is relevant to your study. If you are not sure if a list item applies to your research, read the appropriate section before selecting a response.

Materials & experimental systems

n/a	Involved in the study
[ ]	[x] Antibodies
[ ]	[x] Eukaryotic cell lines
[x]	[ ] Palaeontology and archaeology
[x]	[ ] Animals and other organisms
[x]	[ ] Clinical data
[x]	[ ] Dual use research of concern
[x]	[ ] Plants

Methods

n/a	Involved in the study
[x]	[ ] ChIP-seq
[x]	[ ] Flow cytometry
[x]	[ ] MRI-based neuroimaging

Antibodies

Antibodies used

1. rSC1 Rabbit Derived anti-1pTza antibody (Synthesized in-house)
2. rSC44 Rabbit Derived anti-3-pTza antibody (Synthesized in-house)
3. hSC44 Humanized anti-3pHis Antibodies + Engineered Variants (Synthesized in-house)
4. HRP conjugated mouse anti-M13 (Sino Biological; 11973-MM05T-H)
5. Anti-FLAG antibody (MilliporeSigma; F3165)
6. Goat anti-rabbit IgG (Thermo Scientific™/Invitrogen; A16110)
7. HRP-conjugated goat anti-Human Kappa (SouthernBiotech; 2060-05)
8. Secondary goat anti-rabbit IgG antibody Alexa Fluor® 488 (A-11034, Invitrogen, Carlsbad, CA, USA)
9. Secondary mouse anti-human Ig light chain K antibody Alexa Fluor® 647 (316514, BioLegend®, San Diego, CA, USA)

Validation

1. Validation shown in previous manuscript(s) (doi: 10.1016/j.cell.2015.05.046, doi: 10.1073/pnas.2010644118) as well as this manuscript.
2. Validation shown in previous manuscript(s) (doi: 10.1016/j.cell.2015.05.046, doi: 10.1073/pnas.2010644118) as well as this manuscript.
3. Validation shown in this manuscript.
4. anti-M13 Antibodies (https://cdn1.sinobiological.com/reagent/antibody-application/elisa-protocol-en.pdf)
5. Anti-FLAG Validation - SciCrunch Validation (RRID: AB_439687/AB_262044)
6. Goat anti-rabbit (https://www.thermofisher.com/antibody/product/Goat-anti-Rabbit-IgG-H-L-Highly-Cross-Adsorbed-Secondary-Antibody-Polyclonal/A16110)
7. HRP-conjugated goat anti-human kappa (https://www.southernbiotech.com/goat-anti-human-kappa-hrp-2060-05)
8. Alexa-fluor conjugated goat anti-rabbit IgG (https://www.thermofisher.com/antibody/product/Goat-anti-Rabbit-IgG-H-L-Highly-Cross-Adsorbed-Secondary-Antibody-Polyclonal/A-11034
9. Alexa-fluor conjugated mouse anti-human IgG (https://www.biolegend.com/fr-fr/products/alexa-fluor-647-anti-human-ig-light-chain-kappa-antibody-3301)

Eukaryotic cell lines

Policy information about cell lines and Sex and Gender in Research

Cell line source(s)	Cell Lines were obtained from the American Type Culture Collection (ATCC) with the following accession numbers: (1) SK-N-BE(2) CRL-2271. (2) HEK293T ACS-4500
Authentication	Cell lines were not recently authenticated.
Mycoplasma contamination	Cell lines tested negative for mycoplasma contamination.
Commonly misidentified lines (See ICLAC register)	N/A

Plants

Seed stocks	N/A
Novel plant genotypes	N/A
Authentication	N/A
